# Clathrin-associated AP-1 controls termination of STING signalling

**DOI:** 10.1038/s41586-022-05354-0

**Published:** 2022-10-19

**Authors:** Ying Liu, Pengbiao Xu, Sophie Rivara, Chong Liu, Jonathan Ricci, Xuefeng Ren, James H. Hurley, Andrea Ablasser

**Affiliations:** 1 Global Health Institute, Swiss Federal Institute of Technology Lausanne (EPFL), Lausanne, Switzerland; 2 Department of Molecular and Cell Biology and California Institute for Quantitative Biosciences, University of California, Berkeley, Berkeley, CA USA; 3 Helen Wills Neuroscience Institute, University of California, Berkeley, Berkeley, CA USA

**Keywords:** Innate immunity, Membrane trafficking, Signal transduction

## Abstract

Stimulator of interferon genes (STING) functions downstream of cyclic GMP-AMP synthase in DNA sensing or as a direct receptor for bacterial cyclic dinucleotides and small molecules to activate immunity during infection, cancer and immunotherapy^1–10^. Precise regulation of STING is essential to ensure balanced immune responses and prevent detrimental autoinflammation^11–16^. After activation, STING, a transmembrane protein, traffics from the endoplasmic reticulum to the Golgi, where its phosphorylation by the protein kinase TBK1 enables signal transduction^17–20^. The mechanism that ends STING signalling at the Golgi remains unknown. Here we show that adaptor protein complex 1 (AP-1) controls the termination of STING-dependent immune activation. We find that AP-1 sorts phosphorylated STING into clathrin-coated transport vesicles for delivery to the endolysosomal system, where STING is degraded^21^. We identify a highly conserved dileucine motif in the cytosolic C-terminal tail (CTT) of STING that, together with TBK1-dependent CTT phosphorylation, dictates the AP-1 engagement of STING. A cryo-electron microscopy structure of AP-1 in complex with phosphorylated STING explains the enhanced recognition of TBK1-activated STING. We show that suppression of AP-1 exacerbates STING-induced immune responses. Our results reveal a structural mechanism of negative regulation of STING and establish that the initiation of signalling is inextricably associated with its termination to enable transient activation of immunity.

## Abstract (web summary)

The adaptor protein AP-1 controls the shutdown of STING signalling through a mechanism in which AP-1 recognizes a dileucine motif in phosphorylated STING, which leads to targeted transport of STING to the endolysosomal system for degradation.

## Main

After activation and exit from the endoplasmic reticulum (ER), STING engages two bifurcating cellular effector pathways. The first pathway diverges along STING’s transition to the Golgi and enables autophagy, an ancestral antiviral function of STING; by contrast, the second pathway, which begins at the Golgi, promotes the transcriptional activation of innate immune genes—an evolutionarily more recent functional adaptation^22,23^. Both pathways eventually converge at the lysosome, where STING is degraded^21,23–25^. The initiation of STING’s downstream transcription cascade is controlled by a multi-step process: it begins with the recruitment of TBK1, continues with the phosphorylation of STING by TBK1 and results in the engagement of interferon regulatory factor 3 (IRF3) by phosphorylated STING^17,20,26,27^. STING-bound IRF3, in turn, is phosphorylated by TBK1, and translocates to the nucleus to regulate gene expression jointly with NF-κB and other transcription factors. Through this cascade of molecular events, STING triggers a broad range of effector functions, most notably the expression of type I interferons (IFNs), proinflammatory cytokines and co-stimulatory molecules. Owing to these favourable immunostimulatory properties, STING agonists are being developed for use as immunotherapeutic agents^3,4,28^. However, to optimize robust immune activation while preventing immunopathology, STING responses require strict regulation. Here, we looked for a mechanism that explains how STING-dependent immune signalling is ended.

**Fig. 1:**
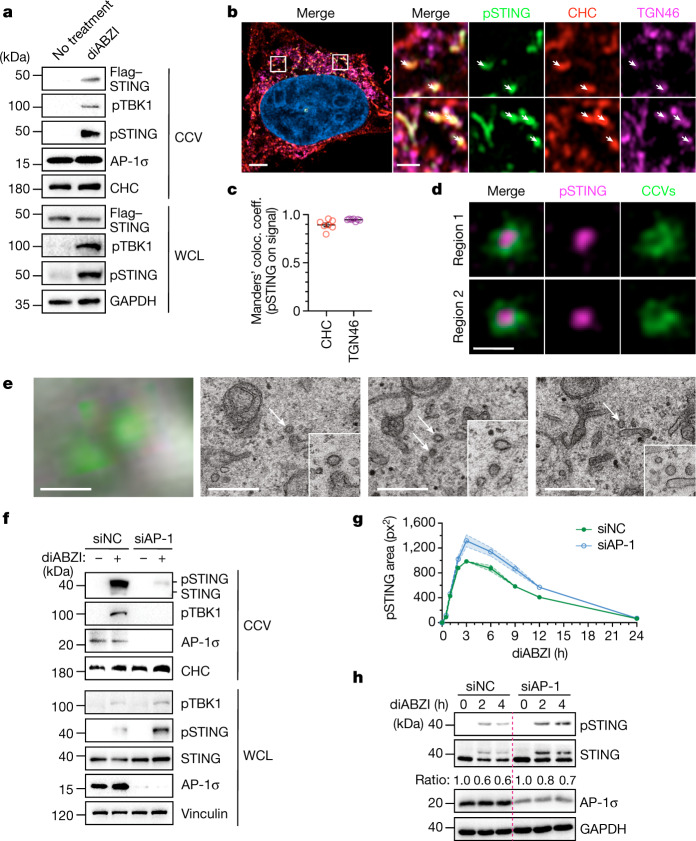
AP-1 loading of STING into CCVs at the TGN. **a**, Whole-cell lysate (WCL) and CCV fractions from HeLa cells expressing Flag-tagged STING (HeLa^STING^) were analysed by western blot. Clathrin heavy chain (CHC) and GAPDH were used as loading and processing controls. **b**, Airyscan imaging of HeLa^STING^ cells stimulated with diABZI. One representative cell of *n* = 7 cells. White arrows point at occurrences of pSTING. Scale bars, 4 µm (left) or 1 µm (magnified panels). **c**, Quantification of the colocalization of pSTING with CHC described in **b**, using Manders’ colocalization coefficients. Mean ± s.e.m. of *n* = 7 cells. **d**, STED images showing pSTING enclosed in CCVs from cells transfected with mCherry–clathrin and Flag–STING and stimulated with diABZI. Regions 1 and 2 are magnified from a large-field-of-view STED image (see Extended Data Fig. 2a). Scale bars, 200 nm. **e**, CLEM of HeLa^GFP–STING^ cells stimulated with diABZI. The images depict box 3 of Extended Data Fig. 2b in Airyscan microscopy (left) or electron microscopy at different *Z*-heights (three right panels). White arrows indicate CCVs. Scale bars, 0.5 µm. **f**, WCL and CCV fractions from HeLa cells that were treated with non-targeting control (NC) small interfering RNA (siRNA) or AP-1 siRNA and stimulated with diABZI were analysed by western blot. CHC was used as a loading control. **g**, Quantification of the area of pSTING in bright-field fluorescent microscopy images of HeLa^STING^ cells that were treated with siRNAs and stimulated with diABZI. Mean ± s.e.m. of *n* = 3 independent experiments with 99 fields of view per condition. **h**, HeLa cells transfected with siRNAs and stimulated with diABZI were analysed by western blot. Ratios of STING versus loading control (GAPDH) normalized to the 0-h time point of each condition. One representative example of three (**a**–**c**,**h**) or two (**f**) independent experiments is shown. Source Data

**Extended Data Fig. 1:**
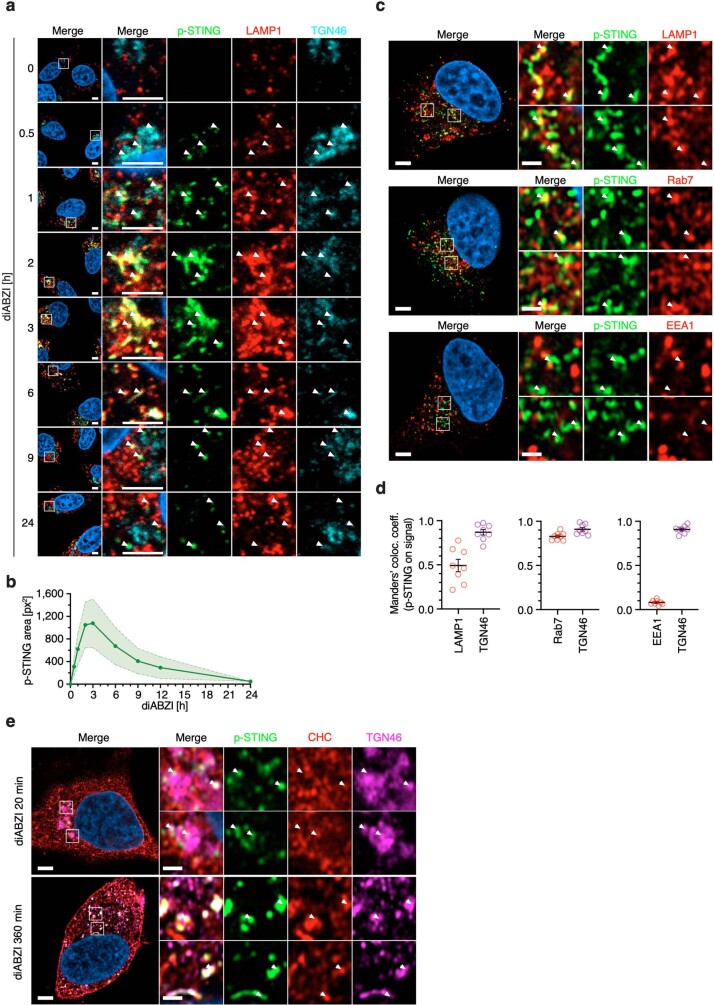
Intracellular trafficking of pSTING. **a**,**b**, Confocal imaging (**a**) or quantification of pSTING area in bright-field fluorescent microscopy images (**b**) of HeLa^STING^ cells stimulated for 0, 0.5, 1, 2, 3, 6, 9, 12 or 24 h with 1 µM diABZI. Cells were fixed and stained for TGN46 (*trans*-Golgi), pSTING and LAMP1 (endolysosomal compartment) as well as with Hoechst dye (nuclei, in dark blue). Mean ± s.e.m. of *n* = 3 independent experiments with at least 84 fields of view each per condition. Scale bars, 4 µm. **c**,**d**, Airyscan imaging (**c**) of HeLa^STING^ cells stimulated for 2.5 h with 1 µM diABZI. Cells were fixed and stained for TGN46 (*trans*-Golgi, not shown here), pSTING and the indicated marker as well as with Hoechst dye (nuclei, in dark blue). Colocalization of pSTING with the indicated marker is quantified by Manders’ colocalization coefficients (**d**). One representative cell is shown, and quantification is the mean ± s.e.m. of *n* = 8 cells examined in 1 of 4 independent experiments. Scale bars, 4 µm in larger left panel, 1 µm in zoomed-in panels. **e**, Airyscan imaging of HeLa^STING^ cells stimulated for 20, 150 (see Fig. 1b) or 360 min with 1 µM diABZI. Cells were fixed and stained for TGN46 (*trans*-Golgi), pSTING and CHC (clathrins) as well as with Hoechst dye (nuclei, in dark blue). One representative cell is shown of at least *n* = 6 cells from 3 independent experiments. White arrows point at occurrences of pSTING. Scale bars, 4 µm in larger left panel, 1 µm in zoomed-in panels. Source Data

**Extended Data Fig. 2:**
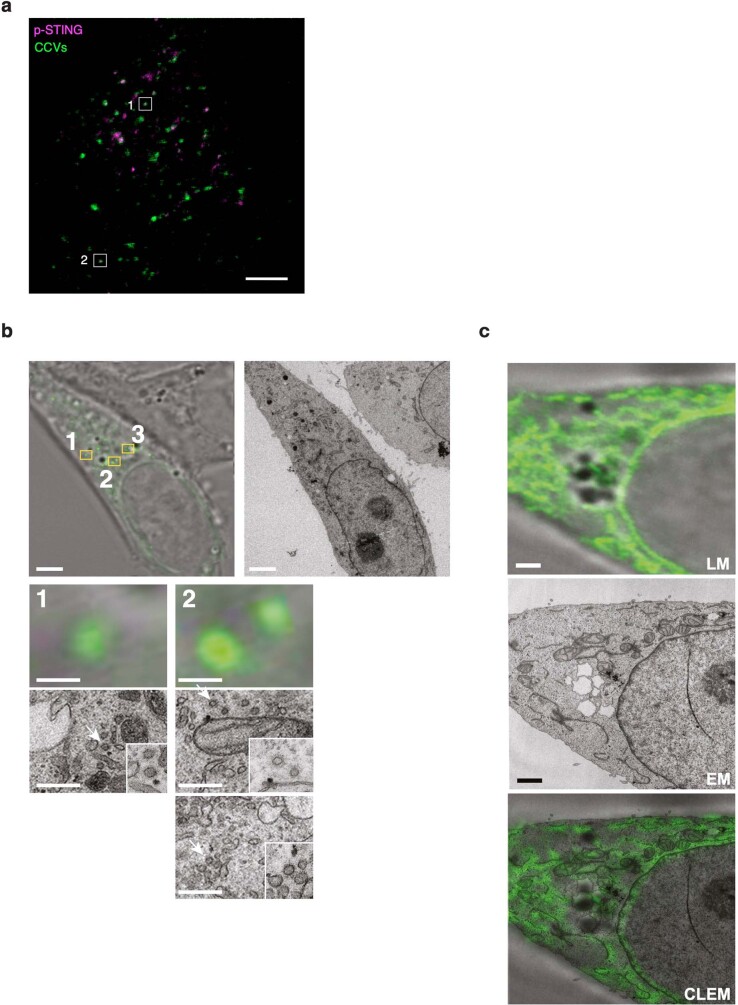
Super-resolution imaging of STING in CCVs. **a**, Large-field-of-view STED image showing the colocalization of pSTING and CCVs. HeLa cGAS/STING double KO cells were transfected with mCherry-clathrin and Flag-STING. One day later they were stimulated with 1 µM diABZI for 2.5 h and then fixed and stained for pSTING. Two representative events highlighted in the boxes were magnified (see Fig. 1d). Scale bar, 2 μm. The image depicts *n* = 1 cell out of 62 cells imaged over the course of 2 independent experiments, including 25 cells showing clear colocalization and 3 exhibiting clathrin ring structures. **b**,**c**, CLEM of HeLa STING KO cells stably reconstituted with GFP-hSTING stimulated for 2.5 h with 1 µM diABZI (**b**) or left untreated (**c**). For the stimulated cell (**b**), some regions with accumulation of high GFP–STING intensity (yellow boxes) were re-imaged by electron microscopy (EM) in higher resolution at relevant Z-heights. Zoom-in and higher-resolution electron microscopy slices from box 3 is shown in Fig. 1e. LM – light microscopy (Airyscan). White arrows indicate CCVs. Scale bars in (**b**), 2 µm (un-zoomed top images) and 0.5 µm (zoom-in box images). Scale bars in (**c**), 1 µm. *n* = 1 out of 3 stimulated cells (**b**) and *n* = 1 out of 2 non-stimulated cells imaged from one sample per condition prepared for CLEM.

We tracked activated STING (phosphorylated at S366; hereafter pSTING)) (ref. ^17^) along its intracellular trafficking route by high-resolution confocal and Airyscan microscopy in HeLa cells. Extending previous findings^20,21^, after stimulation with the small-molecule agonist diABZI-C3 (hereafter, diABZI) (ref. ^3^), pSTING rapidly (around 0.5 h) appeared at the *trans*-Golgi network (TGN) and then moved to LAMP1^+^ endolysosomal compartments before disappearing (Extended Data Fig. 1a–d). pSTING transited RAB7^+^ late endosomes, but did not colocalize with EEA1^+^, a marker of early endosomes (Extended Data Fig. 1c,d). Membrane traffic between the TGN and endocytic organelles can involve clathrin-coated transport vesicles (CCVs)^29^. Of note, STING and pSTING were enriched in CCVs obtained from diABZI-stimulated cells, but not in those obtained from unstimulated cells (Fig. 1a). Furthermore, pSTING colocalized with clathrin heavy chain, a defining component of CCVs, at TGN46^+^ compartments in activated cells (Fig. 1b,c and Extended Data Fig. 1e). Stimulated emission depletion (STED) super-resolution microscopy confirmed that pSTING is incorporated into small (around 100-nm diameter) CCVs (Fig. 1d and Extended Data Fig. 2a). In correlated light and electron microscopy (CLEM) experiments, we observed that, upon activation, characteristic STING perinuclear foci localized to areas that contain multiple CCVs (Fig. 1e and Extended Data Fig. 2b,c). Together, these results show that CCVs can function as transport vehicles of activated STING.

## AP-1 sorts STING into CCVs

**Extended Data Fig. 3:**
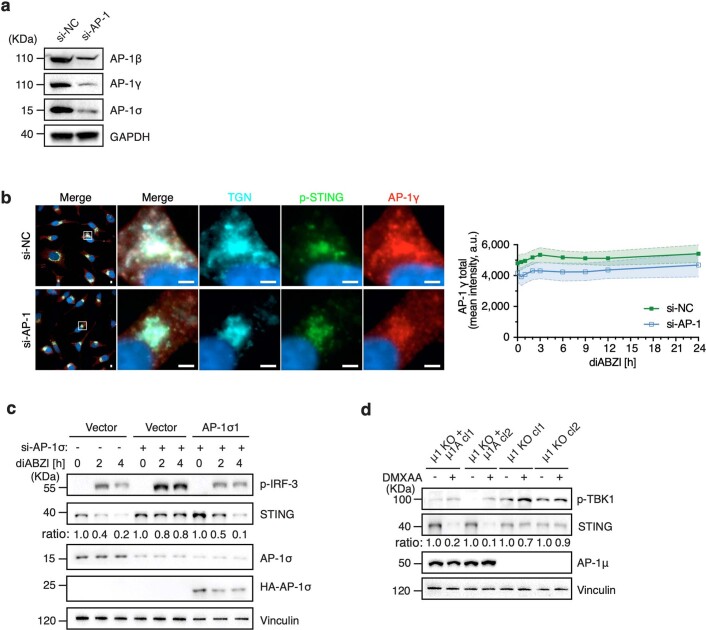
AP-1 associates with STING after activation. **a**, Western blot showing levels of AP-1 subunits after NC siRNA or AP-1 siRNA transfection in HeLa cells. GAPDH was used as a loading control. **b**, Bright-field fluorescent microscopy images and corresponding quantification of AP-1γ signal intensity of HeLa^STING^ cells treated for 3 days with NC siRNA or AP-1 siRNA and then stimulated for 0, 0.5, 1, 2, 3, 6, 9, 12 or 24 h with 1 µM diABZI. Cells were then fixed and stained for TGN46 (*trans*-Golgi), pSTING and AP-1γ as well as with Hoechst dye (nuclei, in dark blue). Images shown here are from the 2-h time points. Mean ± s.e.m. of *n* = 3 independent experiments with 99 fields of view each per condition. Scale bars, 4 µm. **c**, HeLa cGAS KO cells incubated with NC siRNA or siRNAs targeting AP-1σ1 and AP-1 σ3 for 3 days and reconstituted with empty vector or HA-tagged AP-1σ1 were treated with 2.5 µM diABZI for 0, 2, 4 h before analysis by western blot. Vinculin was used as a loading control. **d**, AP-1 μ1 KO MEFs and μ1 KO + μ1A MEFs were treated with 0.5 μg/mL DMXAA for 2 h and analysed by western blot. Vinculin was used as a loading control. One representative of three (**b**,**c**) or at least two (**a**,**c**,**d**) independent experiments is shown. Ratios of target proteins versus loading control normalized to the untreated sample of each condition (**c**,**d**). Source Data

The formation of CCVs relies on heterotetrameric adaptor protein complexes, which physically connect clathrin with transmembrane cargo proteins^29–31^. The most prominent adaptor protein complex that is involved in cargo shuttling from the TGN to endolysosomes is AP-1, which consists of the four subunits β1, γ, µ1 and σ1 (ref. ^32^). Notably, a knockdown of three AP-1 subunits (*AP1G1*, *AP1S1* and *AP1B1*) resulted in a marked reduction in the levels of STING, pSTING and pTBK1 within CCVs, whereas the overall cellular levels of STING and pSTING were increased (Fig. 1f–h and Extended Data Fig. 3a). Crucially, STING trafficking from the ER to the TGN was not affected in AP-1-depleted cells (Extended Data Fig. 3b). We confirmed that a knockdown of the σ1 subunit alone or a genetic knockout of the µ1 subunit in mouse embryonic fibroblasts (MEFs) affected the degradation of STING, and that reintroducing the missing subunit restored the decay of activated STING (refs. ^33,34^) (Extended Data Fig. 3c,d). These findings suggest that AP-1 has a role in controlling activated STING by gating its loading into CCVs.

**Fig. 2:**
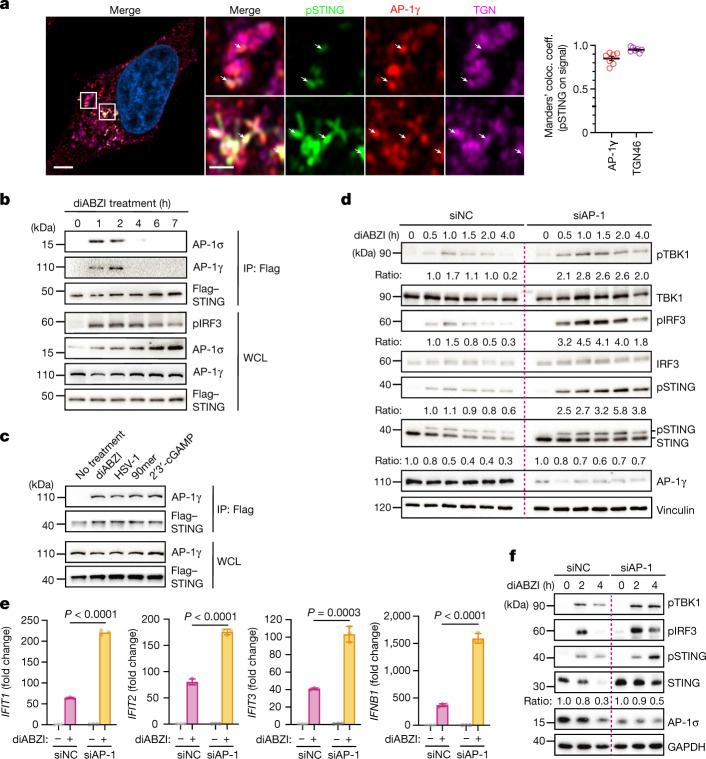
AP-1 binding directs STING degradation to limit immune activation. **a**, Airyscan imaging of HeLa^STING^ cells that were stimulated for 2.5 h with diABZI. The colocalization of pSTING with AP-1γ is quantified by Manders’ colocalization coefficients. One representative cell is shown, and the quantification is the mean ± s.e.m. of *n* = 7 cells from one out of four independent experiments. White arrows point at occurrences of pSTING. Scale bars, 4 µm (left) or 1 µm (magnified panels). **b**, HeLa^STING^ cells were stimulated with diABZI. After immunoprecipitation (IP) with anti-Flag antibody, cells were analysed by western blot. **c**, HeLa^STING^ cells were stimulated with diABZI for 2 h, infected with HSV-1 (multiplicity of infection (MOI) = 10) for 6 h or transfected with 90mer dsDNA (1 µg) for 3 h or 1 µM 2’3’-cGAMP for 6 h. After immunoprecipitation with anti-Flag antibody, samples were analysed by western blot. **d**, HeLa cells transfected with siRNAs were stimulated with diABZI and analysed by western blot. Vinculin was used as a loading control. **e**, Induction of *IFNB1*, *IFIT1*, *IFIT2* and *IFIT3* expression was assessed by quantitative PCR with reverse transcription (RT–qPCR) in HeLa cells transfected with siRNAs and treated with diABZI for 3 h. Ratios of *IFNB1*, *IFIT1*, *IFIT2* and *IFIT3* mRNA versus *GAPDH* mRNA normalized to the untreated groups of each condition. Data are mean ± s.d. of three technical replicates. *P* values were obtained by two-tailed Student’s *t*-test. **f**, WI-38 human fibroblasts transfected with siRNAs for three days were stimulated with diABZI and analysed by western blot. GAPDH was used as a loading control. One representative example of three (**a**,**d**–**f**) or two (**b**,**c**) independent experiments is shown. Ratios of target proteins versus loading control normalized to the 0-h time point of each condition (**d**,**f**). Source Data

**Extended Data Fig. 4:**
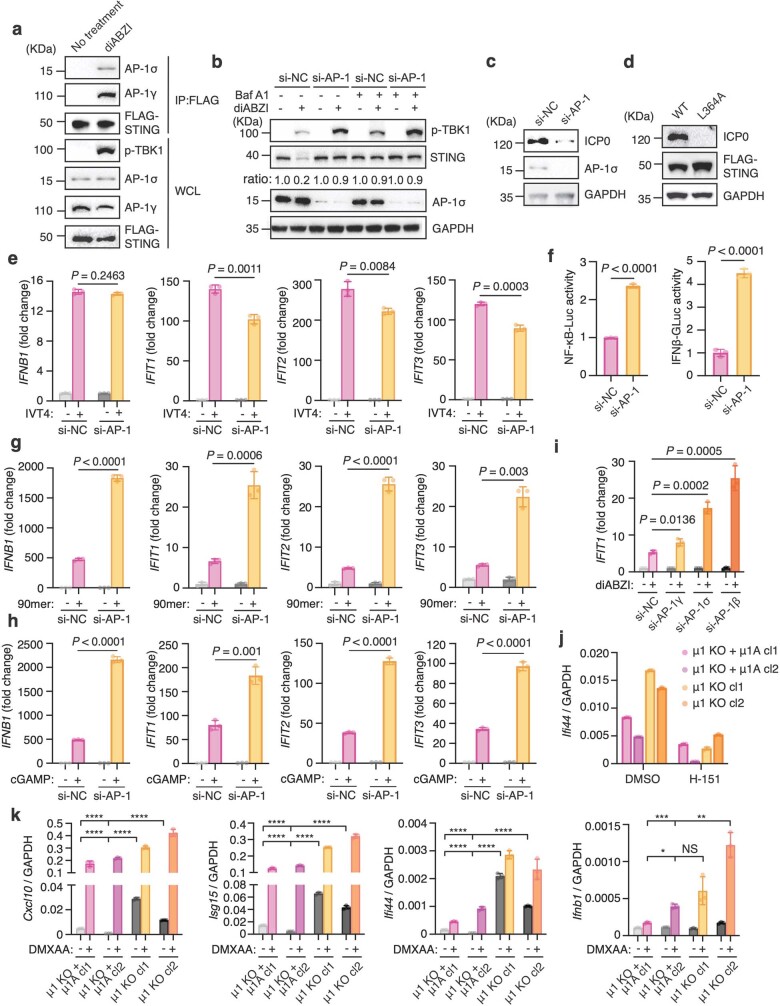
AP-1 depletion boosts STING-dependent immune activation. **a**, HEK293T cells transfected with FLAG-tagged STING^WT^ were treated with 2.5 µM diABZI or left untreated for 2 h, immunoprecipitated (IP) with anti-FLAG antibody, and analysed by western blot. **b**, HeLa cells transfected with NC siRNA or AP-1 siRNA for 3 days were treated with 20nM Baf A1 and 2.5 µM diABZI for 2 h before analysed by western blot. GAPDH was used as a loading control. **c**, HeLa cells incubated with NC siRNA or AP-1 siRNA for 3 days were infected with HSV-1 (MOI = 5) for 14 h and analysed by western blot. GAPDH was used as a loading control. **d**, HeLa cGAS/STING double KO cells transfected with FLAG-tagged STING^WT^, STING^L364A^ were treated with 2.5 µM diABZI for 2 h were infected with HSV-1 (MOI=5) for 14 h and analysed by western blot. GAPDH was used as a loading control. **e**, mRNA levels of *IFNB1*, *IFIT1*, *IFIT2* and *IFIT3* in HeLa cells transfected with NC siRNA or AP-1 siRNA for 3 days and treated with 0.5 μg/mL IVT4 were assessed by RT–qPCR. **f**, NF-kB and IFN-β luciferase assay in HEK293T cells incubated with NC siRNA or AP-1 siRNA for 3 days, followed by transient expression of STING and stimulation with diABZI (2.5 µM). **g**, Induction of *IFNB1*, *IFIT1*, *IFIT2*, *IFIT3* expression was assessed by RT–qPCR in HeLa cells transfected with NC siRNA or AP-1 siRNA for 3 days and then transfected with 1 μg 90mer. **h**, Induction of *IFNB1*, *IFIT1*, *IFIT2*, *IFIT3* expression was assessed by RT–qPCR in HeLa cells transfected with NC siRNA or AP-1 siRNA for 3 days and then transfected with 1 μM 2’3’-cGAMP. **i**, Induction of IFIT1 expression was assessed by RT–qPCR in HeLa cells transfected with non-targeting control (NC) siRNA or AP-1σ, AP-1β and AP-1γ siRNA for 3 days and then treated with 1 µM diABZI for 3 h. **j**, mRNA levels of mouse ifi44 in MEFs cells treated with DMSO or 2 µM H-151 for 3 days were assessed by RT–qPCR. **k**, mRNA levels of mouse *Cxcl10*, *Isg15*, *ifi44*, *ifnb1* in MEFs cells treated with 40 µg/mL DMXAA for 3 h were assessed by RT–qPCR. One representative of three (**b**,**e**–**k**) or at least two (**a**,**c**,**d**) independent experiments is shown. For RT–qPCR experiments, ratios of *IFNB1*, *IFIT1*, *IFIT2*, *IFIT3* mRNA versus *GAPDH* mRNA normalized to the untreated groups of each condition. Mean ± s.d. of three (**e**–**k**) technical replicates. *P* values based on two-tailed Student’s *t-*tests (**e**–**i**,**k**). *P < 0.05, **P < 0.01, ***P < 0.001, ****P < 0.0001 (**k**). Ratios of target proteins versus loading control normalized to the 0 time point of each condition (**b**). Source Data

Adaptor-protein-dependent sorting requires direct engagement between the multimeric complex and its cargo on target membranes^30,31^. Accordingly, pSTING colocalized with AP-1 at the TGN, and STING robustly bound the γ and σ1 subunits of AP-1 after activation (Fig. 2a,b and Extended Data Fig. 4a). STING associated with AP-1 in response to diverse cellular activators of STING, including double-stranded DNA (dsDNA), cyclic GMP-AMP (cGAMP) and the physiological activator herpes simplex virus 1 (HSV-1), providing evidence that AP-1 recognition is an integral element of the cell biological regulation of STING (Fig. 2c).

## AP-1 restricts STING signalling

**Extended Data Fig. 5:**
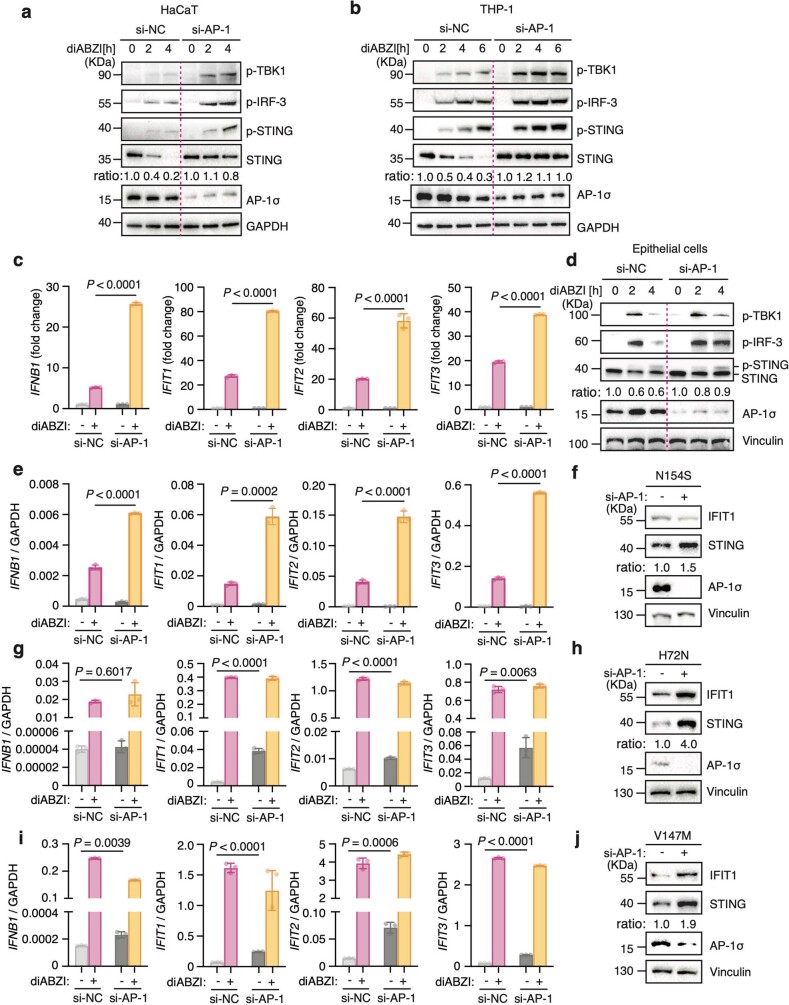
AP-1 depletion boosts STING-dependent immune activation in different cell types. **a**,**b**, HaCaT cells (**a**) or THP-1 cells (**b**) transfected with NC siRNA or AP-1 siRNA for 3 days were treated with 2.5 µM diABZI for indicated time and analysed by western blot. GAPDH was used as a loading control. **c**,**d**, Human primary epithelial cells incubated with NC siRNA or AP-1 siRNA for 3 days were treated with 2.5 µM diABZI and were analysed by RT–qPCR (**c**) and western blot (**d**). Vinculin was used as a loading control. **e**–**j**, Fibroblast cells derived from patients with SAVI characterized by *STING1* N154S mutation (**e**,**f**), H72N (**g**,**h**) or V147M (**i**,**j**) were incubated with NC siRNA or AP-1 siRNA for three days and then analysed by western blot. Vinculin was used as a loading control in **f**,**h**,**j**. **e**,**g**,**i**, Induction of *IFNB1*, *IFIT1*, *IFIT2*, *IFIT3* expression was assessed by RT–qPCR in HeLa cells transfected with NC siRNA or AP-1 siRNA for 3 days and then stimulated with 1 µM diABZI. Mean ± s.d. of three (**c**,**e**,**g**,**i**) technical replicates. *P* values based on two-tailed Student’s *t-*tests (**c**,**e**,**g**,**i**). Source Data

We next determined how the disruption of AP-1 affects STING-dependent immune signalling. A knockdown of AP-1 increased the levels of pTBK1 and pIRF3, in addition to pSTING, and prolonged signalling when compared to stimulated control cells (Fig. 2d). Blocking lysosomal acidification further boosted the activation of TBK1 in AP-1-depleted cells, consistent with a model in which AP-1 functions as the initiator of signalling shutdown upstream of lysosomes (Extended Data Fig. 4b). Cells that were depleted of AP-1 exhibited an increased type I IFN response, which correlated with a more potent inhibition of HSV-1 replication (Fig. 2e and Extended Data Fig. 4c). By contrast, suppression of AP-1 mildly decreased type I IFN responses elicited by triphosphate RNA, a trigger for RIG-I-like helicases, showing that AP-1-mediated negative regulation of innate immune activation is a specific feature of STING signalling (Extended Data Fig. 4e). Analysis of various cells, stimuli and read-outs as well as single subunit knockdowns showed that AP-1-dependent restriction of type I IFN signalling is a universal mechanism for controlling the activity of STING (Fig. 2f and Extended Data Figs. 4f–i and 5a–d). Stronger STING-dependent type I IFN responses were also apparent in µ1A-deficient MEFs as compared to their µ1A-reconstituted counterparts (Extended Data Fig. 4j,k). In STING-associated vasculopathy with onset in infancy (SAVI), gain-of-function alleles of *STING1* cause constitutive type I IFN responses^12,13^. Depletion of AP-1 in fibroblasts from patients with SAVI with distinct hyperactive STING variants^12,35^ led to exaggerated type I IFN signalling and the accumulation of STING (Extended Data Fig. 5e–j). Collectively, these results establish AP-1 as a crucial and conserved mediator in balancing STING responses in various biologically relevant contexts, and suggest that the recruitment of AP-1 to STING is the initiating event in the termination of immune signalling.

## Direct engagement of STING by AP-1

**Fig. 3:**
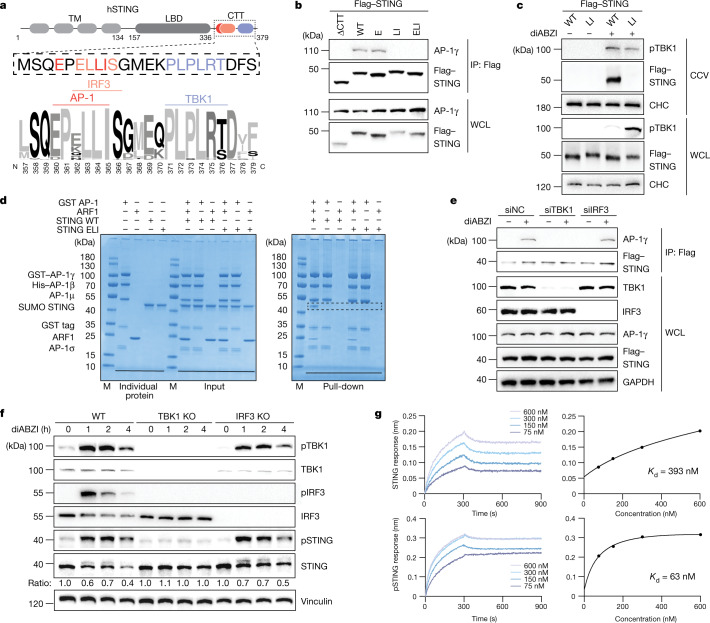
TBK1-dependent phosphorylation controls the binding of STING to AP-1. **a**, Schematic diagram of the CTT of human STING (hSTING) and sequence logo of the CTT as indicated from 50 species. **b**, HeLa STING-knockout (KO) cells transfected with Flag-tagged STING^ΔCΤΤ^ (Δ1–341), wild-type (WT) STING, STING^E^(E360A), STING^LI^(L364A/I365A) or STING^ELI^(E360A/L364A/I365A) were treated with diABZI for 2 h. After immunoprecipitation with anti-Flag antibody, samples were analysed by western blot. **c**, WCL and extracted CCV fractions from HeLa STING KO cells reconstituted with Flag-tagged wild-type STING or STING^LI^(L364A/I365A) and treated or not with diABZI were analysed by western blot. CHC was used as a loading control. **d**, Glutathione sepharose pull-down assays of wild-type LBD-STING or LBD-STING^ELI^ by glutathione *S*-transferase (GST)-tagged AP-1 core with or without ARF1. **e**, HeLa^STING^ cells transfected with NC siRNA or siRNAs against *TBK1* or *IRF3* were treated with or without diABZI. After immunoprecipitation with anti-Flag antibody, samples were analysed by western blot. GAPDH was used as a loading control. **f**, HeLa wild-type cells, HeLa TBK1 KO cells and HeLa IRF3 KO cells stimulated with diABZI for 0, 1, 2 or 4 h were analysed by western blot. Ratios of target proteins versus loading control normalized to the 0-h time point of each condition. Vinculin was used as a loading control. **g**, Bio-layer interferometry binding studies of LBD-STING (top) or TBK1-phosphorylated LBD-STING (pSTING) (bottom) with AP-1 ΔμCTD. The right graphs show the binding affinity of STING (top) and pSTING (bottom). One representative example of at least three (**b**,**d**,**f**) or two (**c**,**e**,**g**) independent experiments is shown. Source Data

**Extended Data Fig. 6:**
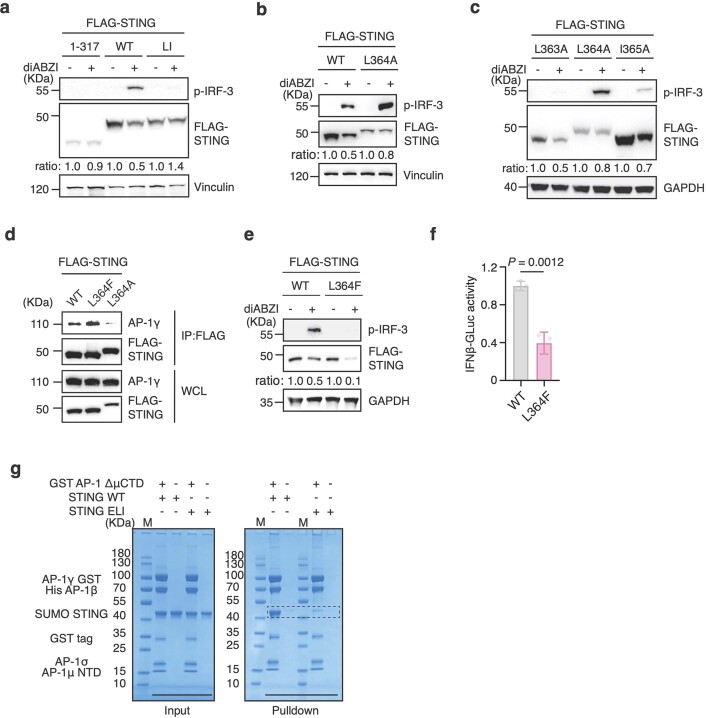
AP-1 binds to STING through a dileucine motif. **a**, HeLa cGAS/STING double KO cells transfected with FLAG-tagged STING^WT^, STING^1–317^ or STING^LI(L364A/I365A)^ were treated or not with 2.5 µM diABZI for 14 h and analysed by western blot. Vinculin was used as a loading control. **b**, HeLa cGAS/STING double KO cells transfected with FLAG-tagged STING^WT^ or STING^L364A^ were treated or not with 2.5 µM diABZI for 14 h and analysed by western blot. Vinculin was used as a loading control. **c**, HeLa cGAS/STING double KO cells transfected with FLAG-tagged STING^L363A^, STING^L364A^ or STING^I365A^ were treated or not with 2.5 µM diABZI for 14 h and analysed by western blot. GAPDH was used as a loading control. **d**, HEK293T cells transfected with FLAG-tagged STING^WT^, STING^L374A^ or STING^L374F^ were treated with 2.5 µM diABZI for 2 h, immunoprecipitated with anti-FLAG antibody, and analysed by western blot. **e**, HeLa cGAS/STING double KO cells transfected with FLAG-tagged STING^WT^ or STING^L364F^ were treated or not with 2.5 µM diABZI for 14 h and analysed by western blot. GAPDH was used as a loading control. **f**, IFN-β luciferase assay in HEK293T cells transfected with plasmids expressing indicated STING constructions and stimulated with diABZI (2.5 µM). **g**, Glutathione Sepharose pull-down assays of LBD-STING^WT^ or LBD-STING^ELI(E360A/L364A/I365A)^ by GST-tagged AP-1 ΔμCTD core. One representative of three (**a**–**c**,**e**–**f**) or two (**d**,**g**) independent experiments is shown. Ratios of target proteins versus loading control normalized to the untreated sample of each condition (**a**–**c**,**e**). Mean ± s.d. of three (**f**) technical replicates. *P* values based on two-tailed Student’s *t-*tests (**f**). Source Data

Adaptor protein interactions depend on the recognition of linear sorting signals in the cytosolic tail of transmembrane cargo proteins^36–38^. Inspection of the cytosolic parts of STING revealed a highly conserved acidic dileucine-based consensus motif [D/E]XXXL[L/I] (in which X denotes any residue) located in the CTT (amino acids 336–379), juxtaposed to the PLPLRT/SD binding motif that is necessary for TBK1 recruitment and partially overlapping with the ɸLXIS recruitment motif (in which ɸ denotes a hydrophilic residue) for IRF3 (refs. ^17–19,27^) (Fig. 3a). Of note, mutations of the two hydrophobic residues disrupted the binding of STING to AP-1 in activated cells, whereas mutation of the acidic residue alone had a negligible effect (Fig. 3b). A STING mutant devoid of the entire CTT also did not bind AP-1 (Fig. 3b). As expected from the requirement of AP-1 for cargo loading, disruption of the LI motif abolished the incorporation of STING into CCVs and compromised subsequent degradation (Fig. 3c and Extended Data Fig. 6a). Moreover, neutralizing the hydrophobic leucine residue at position 364 (EXXXLI) to alanine exaggerated type I IFN signalling, as shown by stronger IRF3 phosphorylation and more potent inhibition of HSV-1 replication (Extended Data Figs. 4d and 6b). Given that the isoleucine motif at position 365 (EXXXLI) is also required for the recruitment of IRF3 onto STING (ɸLXIS)^17,27^, mutagenesis of this position abolished the activation of IRF3 (Extended Data Fig. 6c). Analysing natural variants of STING, we identified a missense mutation, p.LL363LF, located in the EXXXLI motif that has been reported for human cancer^39^. Reconstitution experiments revealed that the L364F substitution increased the binding of STING to AP-1, accelerated STING degradation kinetics and strongly impaired STING signalling activity (Extended Data Fig. 6d–f). Thus, unique substitutions within the EXXXLI motif influence type I IFN activation thresholds of human STING.

To directly analyse the recognition of the STING dileucine motif by AP-1, we reconstituted AP-1 target binding in vitro using the AP-1 core complex^40^. Recombinant STING encompassing the ligand-binding domain (LBD) including the CTT (LBD-STING) robustly interacted with ARF1–GTP-activated AP-1 in pull-down assays^40^, whereas a mutation of the STING dileucine motif (LBD-STING^ELI^) was unable to bind AP-1 (Fig. 3d). Wild-type LBD-STING, but not LBD-STING^ELI^, also bound a version of the AP-1 core in which the µ C-terminal domain is truncated; this variant mimics the open conformation of AP-1 and readily permits interaction with dileucine motifs in the absence of allosteric activation by ARF1 (refs. ^41,42^) (Extended Data Fig. 6g). Together, these results reveal a direct interaction between STING and the ‛unlocked’ activated AP-1 complex, which is mediated by recognition of the STING dileucine motif.

## Phospho-regulation of the binding of STING to AP-1

**Extended Data Fig. 7:**
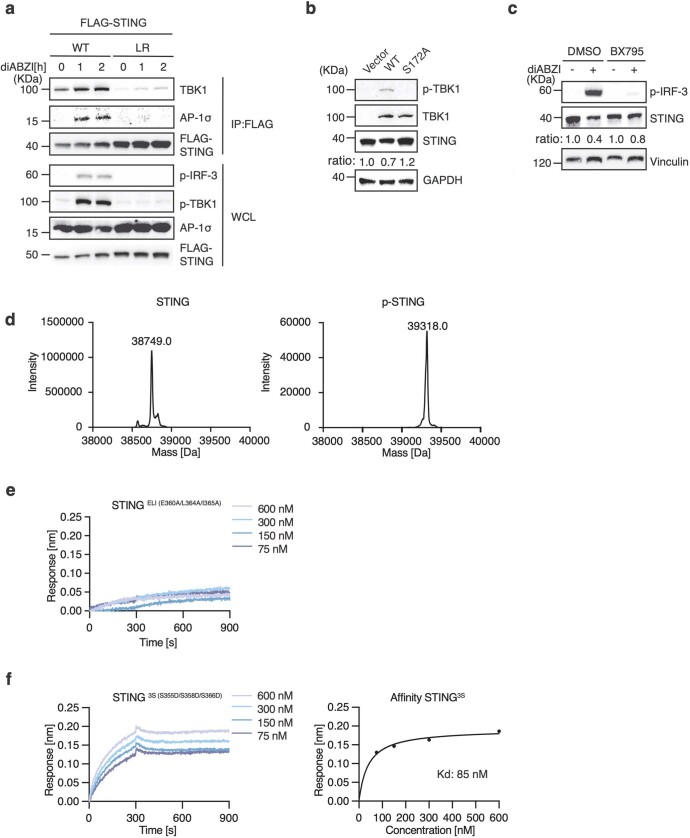
STING–AP-1 interaction depends on TBK1-mediated phosphorylation. **a**, HeLa STING KO cells transfected with FLAG-tagged STING^WT^ or STING^LR(L374A/I375A)^ were treated with 2.5 µM diABZI for 0, 1 or 2 h, immunoprecipitated with anti-FLAG antibody, and analysed by western blot. **b**, HeLa TBK1 KO cells reconstituted with an empty plasmid or with plasmids expressing TBK1^WT^ or enzyme-dead TBK1^S172A^ were treated with 2.5 µM diABZI for 2 h and analysed by western blot. GAPDH was used as a processing control. **c**, HeLa cells pretreated with DMSO or 2 µM BX795 for 24 h were stimulated with 2.5 µM diABZI or not (2 h) and analysed by western blot. Vinculin was used as a loading control. One representative of at least two (**a**–**c**) independent experiments is shown. Ratios of target proteins versus loading control normalized to the untreated sample of each condition (**b**,**c**). **d**, Mass spectrometry detected molecular weight of SUMO LBD-STING and TBK1-phosphorylated LBD-STING (pSTING). **e**, Bio-layer interferometry binding studies of LBD-STING^ELI(E360A/L364A/I365A)^ with AP-1 ΔμCTD. **f**, Bio-layer interferometry binding studies of LBD-STING^3S(S355D/S358D/S366D)^ with AP-1 ΔμCTD. One representative of at least two (**e**, **f**) independent experiments is shown.

Owing to the positioning of the EXXXLI motif relative to the signalling elements for TBK1 and IRF3 (see above; refs. ^17–19^), simultaneous interactions between STING, TBK1, IRF3 and AP-1, respectively, are physically impossible. Thus, we considered whether IRF3 and TBK1 could each interfere with the AP-1 recognition of STING. Whereas depletion of IRF3 had no effect on the binding of STING to AP-1, depletion of TBK1 strongly reduced binding, which suggests that rather than weakening, TBK1 enforces the interaction between STING and AP-1 (Fig. 3e). In agreement with this finding, a truncated version of STING that is defective for TBK1 recruitment (STING^LR^)^18,19^ was unable to bind AP-1 (Extended Data Fig. 7a). In addition, TBK1-knockout cells showed compromised degradation of activated STING, and reconstitution studies and chemical inhibition of TBK1 by BX795 revealed that the effect of TBK1 on STING degradation depends on intact kinase activity (Fig. 3f and Extended Data Fig. 7b,c). We therefore hypothesized that TBK1-mediated phosphorylation might dictate the interaction between STING and AP-1. Quantifying in vitro binding between STING and AP-1 showed that although unmodified LBD-STING bound to the open AP-1 core, in vitro phosphorylation of STING by TBK1 (pLBD-STING) markedly increased the apparent affinity (Fig. 3g and Extended Data Fig. 7d). As expected, LBD-STING^ELI^ showed no binding, whereas a phospho-mimetic STING mutation^18^ increased the binding affinity to a level similar to that seen for in-vitro-phosphorylated STING (Extended Data Fig. 7e,f). Thus, TBK1-dependent phosphorylation is crucial for regulating binding enhancement between STING and AP-1, which in the context of cells is important for efficient cargo recognition.

## Structure of the pSTING–AP-1 complex

**Fig. 4:**
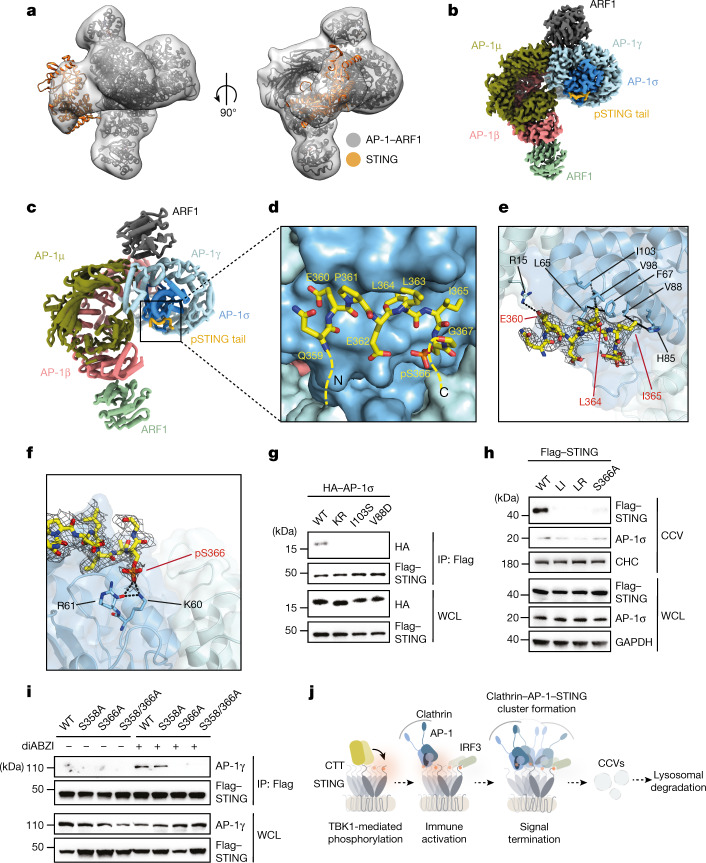
Structural basis for phospho-regulation of STING recognition by AP-1. **a**, Three-dimensional (3D) reconstructions of the complex in two different orientations. The atomic models of dimeric human LBD-STING (Protein Data Bank (PDB) code: 4KSY) and the AP-1 complex (PDB 6DFF) were fitted into the maps (grey) through rigid-body docking. **b**, High-resolution 3D reconstruction from focused refinement on the AP-1 core and pSTING tail at 2.34-Å resolution contoured at 3*σ*. **c**, Ribbon representation of the AP-1 pSTING complex structure. **d**–**f**, Detailed views of the binding interface. **e**, Potentialhydrophobic interactions around the EXXXLI motif are indicated with dotted lines. The density map (grey mesh) of pSTING tail is contoured at 3*σ*. **f**, Potential hydrogen bonds around pS366 indicated with dotted lines. The density map (grey mesh) of the pSTING tail is contoured at 3*σ*. **g**, HEK293T cells transfected with Flag-tagged STING and HA-tagged wild-type AP-1σ or AP-1σ mutants σ^KR^ (K60A/R61A), σ(I103S) and σ(V88D) were stimulated with diABZI for 2 h. Cell lysates were extracted, immunoprecipitated with anti-Flag antibody and analysed by western blot. **h**, WCL and CCV fractions from untreated and diABZI-treated HeLa STING KO cells reconstituted with Flag-tagged wild-type STING and the indicated STING mutants STING^LI^, STING^LR^(L374A/R375A) or STING(S358A) were analysed by western blot. CHC and GAPDH were used as loading controls. **i**, HeLa STING KO cells reconstituted with Flag-tagged STING and the indicated STING mutants STING(S358A), STING(S366A) or STING(S358/366A) were stimulated with diABZI for 2 h or left untreated. After immunoprecipitation with anti-Flag antibody, samples were analysed by western blot. **j**, Schematic diagram of the function of AP-1 in the termination of STING signalling. One representative example of at least three (**g**–**i**) independent experiments is shown.

**Extended Data Fig. 8:**
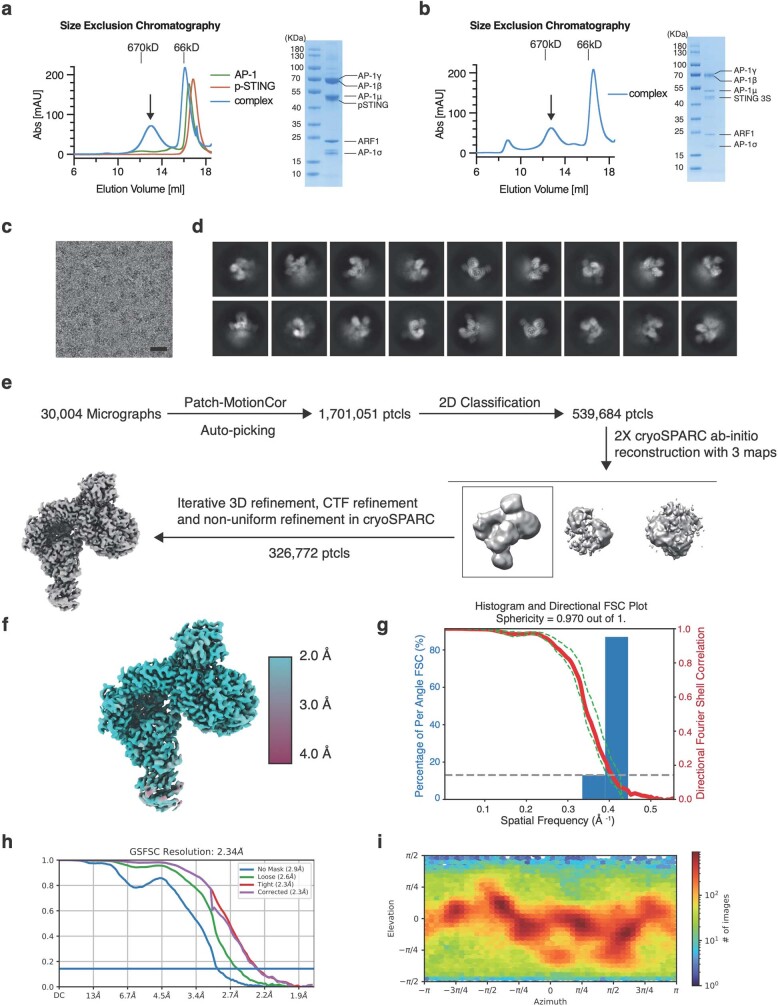
Cryo-EM analysis of pSTING in complex with AP-1. **a**, Purification and SDS–PAGE analysis of AP-1 core in complex with pSTING and ARF1. **b**, Purification and SDS–PAGE analysis of AP-1 core in complex with STING^3S (S355D/S358D/S366D)^. **c**, Representative micrograph of AP-1 pSTING complex in vitrified ice from 30,004 raw images. Scale bar, 30 nm. **d**, 2D class averages of AP-1 pSTING complex particles. Box size, 27 nm. **e**, Flow chart of data processing; see Methods for details.  **f**, Final 3D reconstruction of the AP-1 pSTING complex, coloured according to the local resolution. **g**, 3D Fourier shell correlation of final 3D reconstruction of the AP-1 pSTING complex. **h**, Corrected Gold-standard Fourier shell correlation curves of the AP-1 pSTING complex for the 3D electron microscopy reconstruction. **i**, Angular distribution of the AP-1 pSTING particles included in the final reconstruction. One representative of at least two (**a**,**b**) independent experiments is shown.

**Extended Data Fig. 9:**
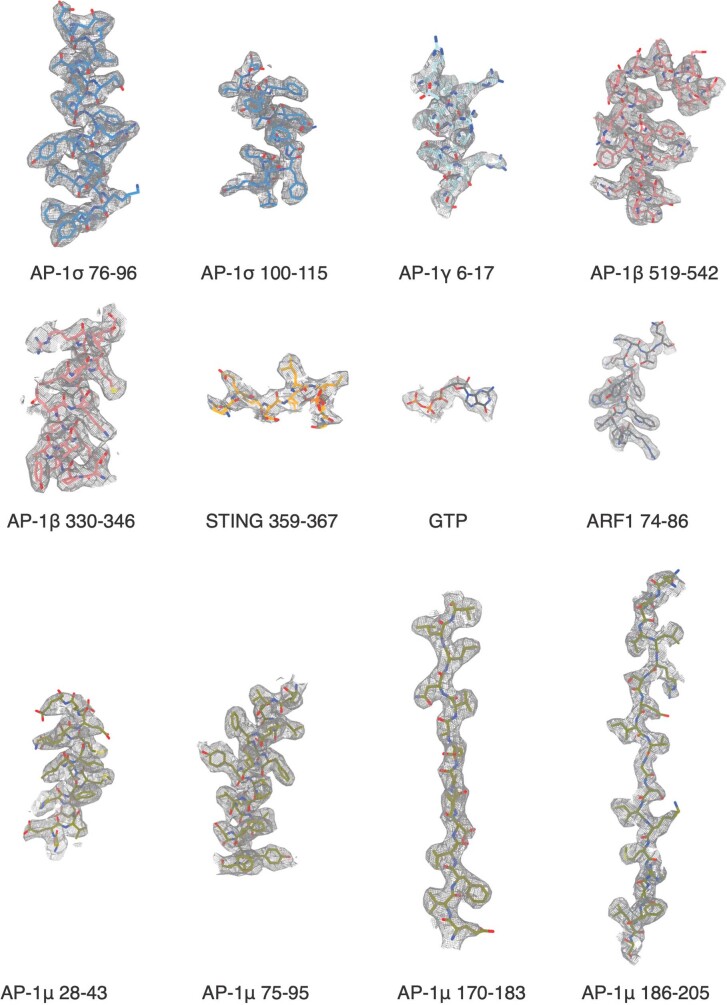
Density maps and structural models of the AP-1–pSTING complex. The density maps (grey mesh) of AP-1 and pSTING contoured at 3σ. The protein structures fitted into the density map are shown by the stick models. Source Data

**Extended Data Fig. 10:**
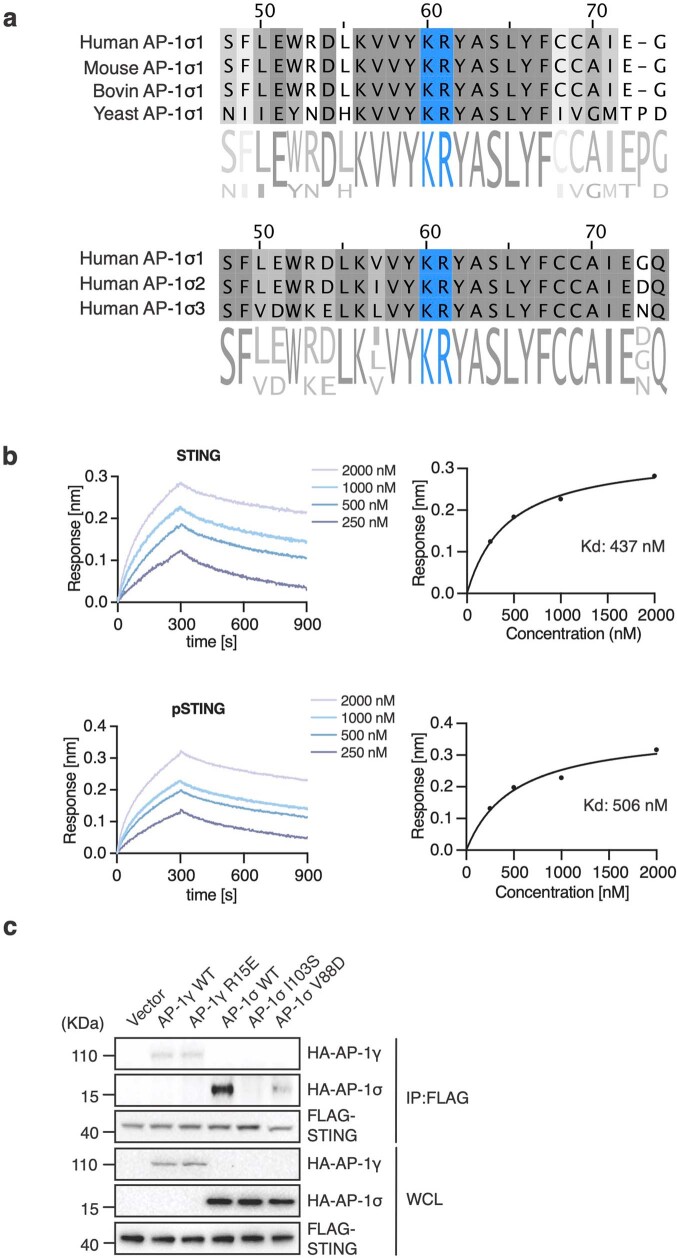
pS366 of pSTING binds to a basic patch of AP-1σ. **a**, Sequence alignment of AP-1σ in different species and human σ subunit isomers. **b**, Bio-layer interferometry binding studies of LBD-STING or pLBD-STING with AP-1 ΔμCTD σ^KR^. **c**, HEK293T cells transfected with FLAG-tagged STING^WT^ and HA-tagged AP-1γ^WT^, γ^R15E^, σ^WT^, σ^I103S^ or σ^V88D^, respectively, were treated with 2.5 µM diABZI for 2 h, immunoprecipitated (IP) with anti-FLAG antibody and then analysed by western blot. One representative of two (**b**,**c**) independent experiments is shown.

To define the mechanism that underlies the enhanced recognition of pSTING by AP-1, we next determined the structure of the complex between the ARF1-activated AP-1 core and TBK1-phosphorylated LBD-STING (Fig. 4a, Extended Data Figs. 8 and 9 and Extended Data Table 1). A 2.3-Å-resolution cryo-electron microscopy (cryo-EM) reconstruction revealed that STING makes multiple contacts with AP-1 through its C-terminal unfolded loop (residues 359–367) (Fig. 4b–f). At the C-terminal part of the loop, STING L364 and I365 of the EXXXLI sorting motif engage L65, F67, H85, V88 and V98 of the σ subunit through hydrophobic interactions (Fig. 4e). At the N-terminal end of the loop, the carboxyl group of E360 contacts R15 of the γ subunit through an electrostatic interaction (Fig. 4e). Together, these contacts anchor STING to AP-1 in a manner similar to that described previously for adaptor protein–cargo peptide complexes^41,43^. Notably, the phospho-moiety on S366 makes hydrogen bonds with a conserved basic patch formed by K60 and R61 of the σ subunit, which can function cooperatively with the dileucine motif interface to enforce binding between pSTING and AP-1 (Fig. 4f and Extended Data Fig. 10a). Binding studies validated that substitution of K60 and R61 selectively reduced the AP-1 binding of pSTING, but not that of native STING, consistent with the importance of these two acidic residues in specifying the recognition of pSTING (Fig. 3g and Extended Data Fig. 10b). In cells, expression of a K60/R61 mutant or substitutions of residues within the σ subunit that are essential for dileucine recognition abolished interaction between STING and AP-1 (refs. ^43,44^) Fig. 4g and Extended Data Fig. 10c). Reciprocally, mutation of S366, but not S358, on STING compromised binding between STING and AP-1 and reduced the sorting of STING into CCVs (Fig. 4h,i). Together, these results show how a unique phosphorylation event within the C terminus of STING confers differential recognition by AP-1, and establish a generalizable mechanism for refining AP-1-based cargo selection through phospho-regulation.

## Discussion

Our results provide insight into a detailed mechanism that underlies the negative-feedback control of STING and, together with previous work^17–19,21^, support a model for TGN-compartmentalized regulation of STING-dependent immune responses (Fig. 4j). First, STING oligomers bind TBK1, which results in robust phosphorylation of STING CTTs to create a platform for interaction with downstream factors. Whereas signal activation is dictated by repeating cycles of IRF3 recruitment, phosphorylation and nuclear translocation, signal inactivation is controlled by AP-1 recognition, which regulates the sorting of activated STING into nascent clathrin coats. Thus, initiation and shutdown of signal transduction at the Golgi are biochemically coupled, thus providing a molecular strategy to self-limit the immune activation induced by STING.

Our structural analysis revealed how remodelling of the primary dileucine binding motif by phosphorylation enables preferential recognition of the activated state of STING by AP-1. Coat formation is a highly cooperative process that depends on the clustering of a large number of discrete ‘adapted’ cargos to efficiently cross-bridge the clathrin triskelion and facilitate vesicle budding. Thus, even a relatively modest gain of affinity in the sorting interface, as provided by phosphorylation, can markedly affect cargo selection at the cell level. Notably, previous work in the context of HIV-1 M-Nef has shown that a single phosphorylation event can function to repress dileucine motif and tetherin downregulation by AP-1 (ref. ^41^). This, when taken together with our findings, highlights a considerable level of adaptability, in which phosphorylation can be harnessed in opposing directions to refine dileucine binding and enable AP-1 to discriminate between distinct cargo states.

Despite high conservation in animals, the CTT only emerged in vertebrate STING to enable IFN-based immunity, which complements primordial antiviral effector functions, including autophagy and NF-κB signalling^22,45,46^. In a similar manner, the AP-1-mediated trafficking process that we describe here, which leads to the elimination of IFN-inducing STING, complements the autophagy-mediated degradation of STING (refs. ^24,25^). The notable conservation of the AP-1 recruitment motif in the STING CTT (Fig. 3a) suggests that, in vertebrates, acquisition of the type I IFN signalling module might have evolved together with its own integrated regulatory system.

In summary, by revealing a mechanism of negative feedback at the TGN, our work fills a gap in knowledge to provide a more complete understanding of STING-dependent immunity, and may offer a conceptual strategy to tune the immunogenic effects of STING for therapeutic interventions.

## Methods

### Cell culture

HeLa (CCL-2) cells were obtained from Sigma-Aldrich. HEK293T cells were a gift from D. Trono, originally purchased from ATCC (SD-3515). THP-1 cells and WI-38 cells were obtained from ATCC. HaCaT cells were obtained from CLS. Primary human alveolar epithelial cells (epithelial cells) were obtained from a commercial supplier (Cell Biologics). MEFs (μ1 KO cells and μ1 KO cells reconstituted with μ1A) were a gift from P. Schu. Primary fibroblast cells from three patients with SAVI were provided by R. Goldbach-Mansky. HeLa, HEK293T, WI-38, HaCaT and primary fibroblast cells were cultured in Dulbecco’s modified Eagle’s medium (DMEM, Thermo Fisher Scientific, 41965039) supplemented with 10% (v/v) heat-inactivated fetal bovine serum (FBS) (Thermo Fisher Scientific, Gibco SKU, 10270106), 100 IU ml^−1^ penicillin–streptomycin (BioConcept, 4-01F00-H), 2 mM l-glutamine (Thermo Fisher Scientific, 25030024) and 1 mM sodium pyruvate (BioConcept, 5-60F00-H) at 37 °C and at atmospheric O_2_ and 5% CO_2_. THP-1 cells were cultured in RPMI 1640 medium (Thermo Fisher Scientific, 21875091) supplemented with 10% FBS, 1× penicillin–streptomycin–l-glutamine (Corning, 30-009-Cl) and 1× 2-mercaptoethanol (Gibco) at 37 °C and at atmospheric O_2_ and 5% CO_2_. MEFs were cultured in DMEM (Thermo Fisher Scientific, 41965039) supplemented with 15% (v/v) heat-inactivated fetal bovine serum (FBS) (Thermo Fisher Scientific, Gibco SKU, 10270106), 100 IU ml^−1^ penicillin–streptomycin (BioConcept, 4-01F00-H) and 1 mM sodium pyruvate (BioConcept, 5-60F00-H) at 37 °C and at atmospheric O_2_ and 5% CO_2_. Primary human alveolar epithelial cells (epithelial cells) were cultured in complete human epithelial cell medium (Cell Biologics, H6621), according to the supplier’s instructions. Cell lines were repeatedly tested for mycoplasma by PCR. No method of cell line authentication was used.

### Plasmids

For CRISPR–Cas9 plasmids, single-guide RNA (sgRNA) targeting TBK1, IRF3, cyclic GMP-AMP synthase (cGAS), AP-1σ1 and STING were designed using the web tool CRISPOR^47^. sgRNAs targeting TBK1, IRF3, cGAS and AP-1σ1 were cloned in a pSpCas9(BB)-2A-Puro (PX459) V2.0 plasmid (Addgene, 62988), whereas sgRNAs targeting STING were cloned in a pSpCas9(BB)-2A-GFP (PX458) plasmid (Addgene, 48138). Both plasmids were gifts from F. Zhang^48^. The pEF-Bos-based STING truncations (1–341 and 1–317) and mutations (E360A, LI364/365AA, ELI360/364/365AAA, LR374/375ΑΑ, L364A, L364F and I365A) were obtained by site-directed mutagenesis. pEF-Bos-human TBK1-Flag-His was a gift from S. Cerboni. TBK1(S172A) was generated by single-amino-acid mutation. pCDNA3-HA γ-adaptin 1(AP1G1) (Addgene, 10712) was purchased from Addgene. pCDNA3-HA-AP1S1 was generated by inserting the coding sequences of AP1S1 flanked by 5’ BamHI and 3’ XhoI sites into the pCDNA3 vector. AP1G1(R15E), AP1S1(I103S) and AP1S1(V88D) were obtained by single-amino-acid mutagenesis. The primers used for plasmid constructions and sgRNA sequences are provided in Supplementary Table 1. Plasmids for NF-κB-Luc (Promega, E8491) were purchased from Promega and those for pIFNβ–GLuc were previously described^8^. All constructs were confirmed by DNA sequencing.

### Stable cell lines

HeLa STING KO cells were obtained from F. Martinon^49^. HeLa^STING^ cells and HeLa^GFP–STING^ cells were generated from HeLa STING KO cells by infection with a pTJ lentiviral vector carrying Flag–STING or GFP–STING, respectively, and a puromycin resistance gene. Cells were selected with puromycin (2 µg ml^−1^). HeLa TBK1 KO cells, HeLa RIF3 KO cells and HaCaT σ1 ΚΟ cells were generated using CRISPR–Cas9 technology. In brief, HeLa cells were plated in six-well culture plates at about 80% confluency and transfected. Per well, 3.5 µl Lipofectamine 2000 (Life Technologies, 11668019) and 1 µg plasmid-DNA were each diluted in 125 µl OptiMEM (Life Technologies, 31985047), mixed, incubated for 5 min and added on top of the well. The next day, the culture medium was replaced and cells were put under puromycin (5 µg ml^−1^) selection for three days. Surviving cells were expended in antibiotic-free medium and sorted by fluorescence-activated cell sorting (FACS) into single clones three days later. Growing clones were characterized by western blotting and selected for the absence of TBK1. HeLa cGAS/STING KO cells were generated using CRISPR–Cas9 technology. HeLa cells were transfected with a pX459-sgcGAS plasmid for 24 h and then selected with puromycin for three days. Surviving cells were expanded in antibiotic-free medium and then transfected with the pX458-sgSTING plasmid in the same way for three days and sorted by FACS. The cells expressing GFP were maintained as HeLa cGAS/STING double KO cells. Growing clones were characterized by western blotting and selected for the absence of cGAS and STING.

### Transfection

For plasmid transfection, cells were transfected with plasmids and Lipofectamine 2000 reagent (Invitrogen, 11668019) (for all imaging experiments) or GeneJuice transfection reagent (Millipore, 70967) (for all other experiments) following the manufacturer’s respective protocols. For the siRNA knockdown, 3 × 10^4^ cells were transfected with Lipofectamine RNAiMAX transfection reagent (Invitrogen, 13778075) and 40 pmol siRNA, following the manufacturer’s protocol, followed by three days of incubation. Medium containing transfection reagents was replaced with fresh medium 6 h after transfection. Silencer select predesigned siRNAs (4390847), siAP1G1 (s1143), siAP1B1 (s1141), siAP1S1 (s3115) and siAP1S3 (s43490) were purchased from Thermo Fisher Scientific; siIRF3 and siTBK1 were synthesized by Mircosynth. The sequences of siRNAs are provided in Supplementary Table 1.

### Stimulation of cells

Cells were treated with 2.5 μM diABZI (Selleckchem, S8796) and collected at the indicated time points. For cGAMP, 90mer and IVT4 stimulation, 0.1 μM cGAMP (Invivogen), 0.2 μg 90mer or 0.5 μg IVT4 was transfected using Lipofectamine 2000 (Invitrogen, 11668019) according to the manufacturer’s protocol, and cells were incubated for 3 h. The DNA sequences of 90mer is provided in Supplementary Table 1. Poly(I:C) (Invivogen) was added to the cell medium at a final concentration of 10 μg ml^−1^ for 24 h. After infection with the HSV-1 KOS strain (MOI of 10), infected cells were incubated for 6 h. Pretreatment with BX795 (MedChemExpress) was performed at 2 µM for 24 h and pretreatment with bafilomycin A1 (Baf A1; Sigma) was performed at 20 nM for 1 h. MEFs treated with 5 μg ml^−1^ or 40 μg ml^−1^ DMXAA (Invivogen) were collected 2 h or 3 h after stimulation. For STING inhibition by H-151, H-151 (2 µM) was added into cells every 24 h for three days before cells were examined by RT–qPCR.

### Antibodies

Primary antibodies used: mouse monoclonal anti-vinculin (hVIN-1) (Sigma-Aldrich, V9264, immunoblot 1:5,000), rabbit monoclonal anti-GAPDH (14C10) (Cell Signaling Technology, 2118, immunoblot 1:3,000), mouse monoclonal anti-Flag (M2) (Sigma-Aldrich, F1804, immunoblot 1:3,000), rabbit monoclonal anti-human phospho-STING (Ser366) (D7C3S) (Cell Signaling Technology, 19781, immunoblot 1:3,000), rabbit monoclonal anti-phospho-TBK1/NAK (Ser172) (D52C2) (Cell Signaling Technology, 5483, immunoblot 1:1,000), rabbit monoclonal anti-phospho-IRF3 (Ser386) (EPR2346) (Abcam, ab76493, immunoblot 1:1,000), rabbit monoclonal anti-TBK1/NAK (D1B4) (Cell Signaling Technology, 3504, immunoblot 1:1,000), rabbit polyclonal anti-TMEM173/STING (Proteintech, 19851-1-AP, immunoblot 1:1,000), rabbit monoclonal anti-IRF3 (D6I4C) (Cell Signaling Technology, 11904, immunoblot 1:1,000), rabbit monoclonal anti-clathrin heavy chain (P1663) (Cell Signaling Technology, 2410, immunoblot 1:500), mouse anti-clathrin heavy chain monoclonal antibody (X22) (Thermo Fisher Scientific, MA1-065, immunofluorescence (IF) 1:100), rabbit polyclonal anti-AP1S1 (Thermo Fisher Scientific, PA5-63913, immunoblot 1:1,000), rabbit polyclonal anti-AP1G1 (Thermo Fisher Scientific, PA5-65290, immunoblot 1:1,000), rabbit polyclonal anti-AP1B1 (Sigma-Aldrich, HPA065226, immunoblot 1:1,000), rabbit polyclonal anti-AP1M1 (Proteintech, 12112-1-AP, immunoblot 1:1,000), mouse monoclonal anti-HSV-1 ICP0 (11060) (Santa Cruz, sc-53090, immunoblot 1:500), mouse monoclonal anti-HA.11 epitope tag (16B12) (Biolegend, MMS-101R, immunoblot 1:2,000). mouse monoclonal γ-adaptin (AP1G1) (100/3) (Sigma-Aldrich, A4200, IF 1:100), mouse monoclonal EEA1 (E9Q6G) (Cell Signaling, 48453, IF 1:100), mouse monoclonal LAMP1 (H4A3) (Abcam, ab25630, IF 1:100), rabbit monoclonal anti-human phospho-STING (Ser366) (D8K6H) (Cell Signaling Technology, 40818, IF 1:100, STED 1:50), mouse monoclonal RAB7 (E9O7E) (Cell Signaling Technology, 95746, IF 1:100) and sheep polyclonal human TGN46 (Bio-Rad, AHP500G, IF 1:200). HRP-conjugated secondary antibodies used: donkey anti-rabbit IgG (H+L)-HRP (Jackson ImmunoResearch, 711-036-152, immunoblot: 1:5,000) and donkey anti-mouse IgG (H+L)-HRP (Jackson ImmunoResearch, 715-036-151, immunoblot: 1:5,000). Fluorescence-conjugated secondary antibodies used: goat anti-mouse IgG2a cross-adsorbed secondary antibody, Alexa Fluor 647-conjugated (Invitrogen, A-21241, IF 1:800), donkey anti-sheep IgG (H+L) cross-adsorbed secondary antibody, Alexa Fluor 488-conjugated (Invitrogen, A-11015, IF 1:800), goat anti-rabbit IgG (H+L) cross-adsorbed secondary antibody, Alexa Fluor 568-conjugated (Invitrogen, A-11011, IF 1:800) and goat anti-rabbit IgG F(ab) ATTO647N (H+L) (Hypermol,2318, IF 1:500). Antibody details are provided in Supplementary Table 1.

### RT–qPCR

Cells were lysed in the RLT buffer (Qiagen). RNA was extracted following the manufacturer’s protocol (Qiagen RNeasy Plant Mini Kit). RNA was reverse-transcribed using the RevertAid First Strand cDNA synthesis Kit (Thermo Fisher Scientific) and analysed by RT–qPCR in triplicate or quadruplicate using the ChamQ Universal SYBR qPCR Master Mix (Vazyme). The qPCR reactions were run on a QuantStudio 7 Real-Time PCR system (Thermo Fisher Scientific). *GAPDH* was used as a housekeeping gene for normalization. Primer sequences are provided in Supplementary Table 1.

### Western blotting and immunoprecipitation

Cells were collected, quickly rinsed with 1× phosphate-buffered saline (PBS) and lysed in lysis buffer (20 mM Tris pH 7.4, 0.5% Triton X-100, 150 mM NaCl, 1.5 mM MgCl_2_, 2 mM EGTA, 2 mM DTT and 1× cOmplete Protease Inhibitor Cocktail (Roche)) on ice for 30 min and centrifuged at 12,000 rpm, 4 °C for 10 min. Supernatants were boiled with 4× loading buffer (200 mM Tris pH 6.8, 8% SDS, 40% glycerol, 0.4 M DTT, 0.4% bromophenol blue) for 10 min. Proteins were resolved by SDS–PAGE using SurePAGE precast gels (GenScript) and transferred to nitrocellulose membranes using the Trans-Blot Turbo RTA Midi Nitrocellulose Transfer Kit (Bio-Rad) following the manufacturer’s instructions. Membranes were blocked with 2% bovine serum albumin (BSA) + 1% milk in PBST (PBS + 0.05% Tween-20) at room temperature for 1 h and then incubated with the primary antibody (diluted in PBST) at 4 °C overnight. After washing in PBST, membranes were incubated with the secondary antibody at room temperature for 1 h. Membranes were washed with PBST, visualized with western blotting detection reagent (Advansta), and imaged using the ChemiDoc XRS Bio-Rad Imager and Image Lab Software. Band intensities were quantified using Fiji software (NIH). For immunoprecipitation, cells were seeded into six-well plates and were transfected with the indicated plasmids. Sixteen hours after transfection, cells were lysed in lysis buffer on ice for 30 min and centrifuged at 12,000 rpm at 4 °C for 10 min. Supernatants were transferred into new tubes and mixed with anti-Flag M2 magnetic beads (Sigma-Aldrich, M8823) at 4 °C overnight on a rotator. After three to six washes with lysis buffer and one to two washes with cold 1× PBS, the beads were boiled with 1× loading buffer for 10 min. Samples of 20 μl were loaded into gel after a short centrifugation, and this was followed by SDS–PAGE and immunoblotting analysis.

### Luciferase assay

HEK293T cells were plated into 96-well plates and transfected with non-targeting control or the combination of siRNAs targeting *AP1G1*, *AP1S1* and *AP1B1* for three days. Cells were transfected using GeneJuice transfection reagent (Millipore) with an IFNβ promoter–reporter plasmid (pIFNβ-GLuc) in combination with a STING-expressing plasmid (pEF-Bos-Flag-STING). Sixteen hours after transfection, cells were stimulated with fresh medium containing 2.5 μM diABZI for 6 h. Gaussia luciferase activity was measured in the supernatants using coelenterazine (PJK GmbH) as substrate. For the measurement of NF-κB promoter luciferase activity, cells were transfected with siRNAs as described before and then transfected using GeneJuice with a NF-κB promoter–reporter plasmid (NF-κB-Luc). After 16 h, cells were stimulated with fresh medium containing 2.5 μM diABZI for 18 h. The promoter activity was determined using the Bright-Glo Luciferase Assay System (Promega). The expression of proteins was confirmed by immunoblotting. For determining the number of viable cells in culture, cells were seeded into a 96-well plate and were measured with CellTiter- Glo Luminescent Cell Viability Assay System (Promega) every 24 h following the producer’s instructions.

### CCV extraction

CCV extraction was performed according to a previously described protocol^50^. In brief, cells from one confluent 500-cm^2^ dish were treated with 2.5 μM diABZI for 2 h and then rinsed with PBS twice. Cells were scraped into 5 ml buffer A (0.1 M MES, pH 6.5, 0.2 mM EGTA, 0.5 mM MgCl_2_) and homogenized by pipetting up and down more than 25 times using a 5-ml syringe with a 22-G needle attached. Cell lysates were centrifuged at 4,100*g*, 4 °C for 32 min. Supernatants were moved into new tubes and treated with 50 μg ml^−1^ ribonuclease A on ice for 30 min followed by centrifugation at 50,000 rpm, 4 °C for 30 min using a Type 70 Ti rotor (Beckman Coulter). Pellets were resuspended in 300 μl buffer A and mixed with an equal volume of buffer B (12.5% (w/v) Ficoll, 12.5% (w/v) sucrose in buffer A), followed by centrifugation at 20,000 rpm, 4 °C for 25 min. Supernatants were transferred into new tubes and diluted with four volumes of buffer A, and centrifuged at 40,000 rpm, 4 °C for 30 min to obtain the CCV-enriched fraction.

### Sample preparation for confocal microscopy

HeLa^STING^ cells were plated in CellCarrier-96 Ultra Microplates (PerkinElmer, 6055302) at a density of 10,000 cells per well and left for at least 5 h to adhere. Cells were then stimulated by adding diABZI at 1 µM (MedChemExpress, HY-103665) for the indicated amount of time. In time-course experiments, cells were stimulated in a sequential manner and fixed all at the same time. At the end of the stimulation, the wells were washed once with PBS, and then cells were fixed by adding paraformaldehyde 4% in CBS buffer (10 mM MES pH 6.9, 138 mM KCl, 2 mM MgCl_2_ and 2 mM EGTA) for 5–10 min at room temperature. Cells were then washed three times for at least 5 min in PBS before blocking for 1–2 h at room temperature with PBS supplemented with 0.1% (v/v) saponin and 5% (v/v) heat-inactivated FBS and later incubating overnight at 4 °C with the primary antibodies diluted in staining solution (PBS supplemented with 0.1% (v/v) saponin) and 1% (w/v) BSA (Sigma-Aldrich, A7906)). Antibodies and concentrations are listed in Supplementary Table 2. The cells were then washed with PBS three times for 5 min and incubated for 1.5 h at room temperature in secondary antibodies diluted in staining solution. From then on, the plate was protected from light. Cells were washed twice more (5 min each) in PBS and incubated for 30–60 min in Hoechst 33342 (Sigma-Aldrich, B2261) 0.2 µg ml^−1^ in PBS. Cells were then put back into 100 µl PBS per well and either imaged directly or kept at 4 °C until imaging. For experiments with KD of AP-1, HeLa^STING^ cells were plated (60,000 cells per well in a six-well plate) and leftt to adhere for 6–16 h. They were then transfected with siRNAs using Lipofectamine RNAiMax reagent (Thermo Fisher Scientific) according to the manufacturer’s instructions. Silencer select predesigned siRNAs were purchased from Thermo Fisher Scientific: negative control (4390847), or siAP1G1 (s1143), siAP1B1 (s1141) and siAP1S1 (s3115); see details in Supplementary Table 1. A total of 2 µl at 10 µM of siRNAs (equally split between the three siRNAs for the siAP-1 wells) combined with 7 µl of lipofectamine RNAiMax reagent in 400 µl OptiMEM total was used per well. The medium was changed after 6–16 h. Three days after siRNA treatment, cells were replated into microscopy plates (10,000 cells per well) and the experiments were continued as described above for non-siRNA-treated cells.

### Imaging and analysis for confocal microscopy

Fixed and stained 96-well plates were then imaged on two microscopes: a confocal Leica SP8 inverted microscope equipped with an HC PL APO 63×/1.40/oil (magnification/N.A./immersion) objective and HyD detectors, operated with the Leica LAS X software; and a PerkinElmer Operetta CLS operated with the PerkinElmer Harmony software and equipped with an Andor Zyla 5.5 camera, and with an LD C Apochromat objective with magnification/N.A./immersion of 63×/1.4/water. Image analysis and quantification were performed by combining PerkinElmer Harmony (v.4.9) and Fiji (v.2.3.0), and data were further processed with KNIME (v.4.3.2) and GraphPad PRISM 9 (v.9.3.1). The panels of images were assembled using OMERO (v.5.11.0)^51^. In more detail, all depicted cell images were acquired with the Leica SP8 confocal microscope (63×), except for the images confirming that pSTING still accumulates at the TGN after treatment with siRNA against AP-1 (compared to siNC), which are images captured on the Operetta (63×). Quantifications of the total area of pSTING or AP1G1 intensity were calculated in Harmony software on the basis of Operetta (63×) images.

### Airyscan microscopy

HeLa^STING^ cells were plated in µ-Slide eight-well chamber slides (ibidi, 80826-IBI) at a density of 10,000 cells per well and left to adhere overnight. Cells were then stimulated by adding diABZI at 1 µM (MedChemExpress, HY-103665) for 0, 150 or 360 min (Fig. 1b depicts the 150-min time point and Extended Data Fig. 1e the 0- and 360-min ones) or only 0 and 150 min (Fig. 2a and Extended Data Fig. 1c,d). In time-course experiments, cells were stimulated in a sequential manner and fixed all at the same time. At the end of the stimulation, the wells were washed once with PBS, and then cells were fixed by adding paraformaldehyde 4% in CBS buffer (10 mM MES pH 6.9, 138 mM KCl, 2 mM MgCl_2_, 2 mM EGTA) for 5–10 min at room temperature. Cells were then washed three times for at least 5 min in PBS before blocking for 1–2 h at room temperature with PBS supplemented with 0.1% (v/v) saponin and 5% (v/v) heat-inactivated FBS and later incubating overnight at 4 °C with the primary antibodies diluted in staining solution (PBS supplemented with 0.1% (v/v) saponin) and 1% (w/v) BSA (Sigma-Aldrich, A7906)). Antibodies and concentrations are listed in Supplementary Table 2. The cells were then washed with PBS three times for 5 min and incubated for 1.5 h at room temperature in secondary antibodies diluted in staining solution. From then on, the plate was protected from light. Cells were washed twice more (5 min each) in PBS and incubated for 30–60 min in Hoechst 33342 (Sigma-Aldrich, B2261, blue on the depicted images) 0.2 µg ml^−1^ in PBS. Cells were then put back into 200 µl PBS per well and either imaged directly or kept at 4 °C until imaging. Imaging was performed with a Zeiss LSM 980 Inverted microscope (multi-purpose confocal with 32 Channels AiryScan, tPMT, widefield and bright-field capability) using the Plan-Apochromat 63×/1.40/oil (magnification/N.A./immersion) objective and the AiryScan mode and images were processed in the ZEN software using embedded AiryScan processing (3D-mode and 'Normal' resolution). Image analysis and quantifications were performed with Fiji (v.2.3.0). Colocalization analysis were performed on Fiji using the BIOP JACoP plug-in with Otsu thresholding for all channels. Data were further processed with KNIME (v.4.3.2) to combine them and remove cells with threshold values lower than 500 for pSTING (considered background), and the results were then plotted with GraphPad PRISM 9 (v.9.3.1). The panels of images were assembled using OMERO (v.5.11.0) (ref. ^51^).

### CLEM

HeLa^GFP–STING^ cells were plated in glass-bottomed Petri dishes (MatTek, P35G-1.5-14-CGRD) with an alpha-numeric grid pattern at a density of 100,000 cells per dish and left to adhere overnight. They were stimulated by adding diABZI at 1 µM (MedChemExpress, HY-103665) for 2.5 h. They were then chemically fixed with a buffered solution of 1% glutaraldehyde and 2% paraformaldehyde in 0.1 M phosphate buffer at pH 7.4. The dishes were then screened with light microscopy to identify cells of interest, which were imaged with both transmitted and fluorescence microscopy (AiryScan mode as described above) to record their position on the grid. The cells were then washed thoroughly with cacodylate buffer (0.1 M, pH 7.4), and post-fixed for 40 min in 1.0% osmium tetroxide with 1.5% potassium ferrocyanide and then 40 min in 1.0% osmium tetroxide alone. They were finally stained for 40 min in 1% uranyl acetate in water before being dehydrated through increasing concentrations of alcohol and then embedded in Durcupan ACM resin (Fluka). The dishes were then filled with 1 mm of resin and this was hardened for 18 h in a 65 °C oven. Cells of interest were then identified according to their position on the alpha-numeric grid, cut away from the rest of the material and glued to a blank resin block. Ultra-thin (50-nm-thick) serial sections were cut through the entire cell with a diamond knife (Diatome) and ultramicrotome (Leica Microsystems, UC7), and collected onto single slot grids with a pioloform support film. These sections were further contrasted with lead citrate and uranyl acetate and images taken in a transmission electron microscope (FEI Company, Tecnai Spirit) with a digital camera (FEI Company, Eagle). To correlate the light microscopy images with the electron microscopy images and identify the exact position of the Centrin-1:GFP foci, fluorescent images were overlaid onto the electron micrographs of the same cell using Photoshop (Adobe).

### STED

HeLa cGAS/STING double KO cells were plated in 3.5-cm glass-bottom Petri dishes (FluoroDish, WFD35-100) at a density of 100,000 cells per dish and left to adhere overnight. They were stimulated by adding diABZI at 1 µM (MedChemExpress, HY-103665) for 2.5 h. They were then transfected with plasmids containing Flag-hSTING and mCherry-clathrin (both in pEF-Bos mammalian expression plasmid) using Lipofectamine 2000 Reagent (Invitrogen, 11668019) according to the manufacturer’s instructions. One microgram per plasmid and 4.5 µl of lipofectamine in 250 µl of OptiMEM (Life Technologies, 31985047) were used for one dish. The medium was replaced with fresh culture medium six hours after transfection. The next day, the cells were stimulated by adding diABZI at 1 µM (MedChemExpress, HY-103665) for 2.5 h. They were then fixed and stained for pSTING as described for the AiryScan microscopy samples. STED images were acquired using a Leica SP8 STED 3X (Leica Microsystems) equipped with a pulsed white light laser (WLL) as an excitation source and a 775-nm pulsed laser as a depletion light source both for mCherry and ATTO647N. Samples were imaged with a 100× objective (Leica, HC APO CS2 100×/1.40/oil) using the LAS X software (Leica Microsystems). For excitation of the respective channels, the WLL was set to 587 nm for mCherry and 647 nm for ATTO647N. Hybrid spectral detectors were used to acquire the images with a final pixel size of 9.2 × 9.2 nm. The detector time gates were set to 1.5–7.5 ns for mCherry and 0.5–6 ns for ATTO647N. Images were acquired as single planes of 1,392 × 1,392 pixels, 600 lines per second, 32× line averaging for mCherry and 16× line averaging for ATTO647N. Deconvolution of STED images was done with Huygens Remote Manager v.3.7, using Good’s roughness maximum likelihood estimation with 60 iterations and a signal-to-noise ratio equal to 2 until it reached a quality threshold of 0.03.

### Protein expression and purification

6His-TEVsite-Hs ARF1 (17-181)-Q71L in pHis2 vector was expressed in BL21 (DE3) bacteria (Sigma-Aldrich, CMC0014). A single colony was inoculated in a culture flask with 100 ml LB with Ampicillin (100 µg ml^−1^) and incubated with shaking (200 rpm, Infors-HT Multitron) at 37 °C overnight as preculture. Large-scale expression of the protein was started the next day by pouring 100 ml of preculture in a 5-l Erlenmeyer flask containing 2 l LB with ampicillin (100 µg ml^−1^). The cells were grown until the optical density at 600 nm reached 0.7. Expression was then induced by adding isopropyl β-d-1-thiogalactopyranoside (IPTG) to a final concentration of 0.5 mM while transferring the culture to an 18 °C shaking incubator overnight. The bacteria were then collected by centrifugation, solubilized in HisTrap buffer A (20 mM HEPES, 500 mM NaCl, 20  M imidazole, 1 mM DTT and 5% glycerol, pH 7.5) supplemented with 4-(2-aminoethyl)-benzolsulfonylfluoride-hydrochloride (AEBSF) and cOmplete protease inhibitors (Roche), lysed by sonication, cleared by centrifugation at 20,000*g* and then passed through a 5-ml nickel immobilized metal-affinity chromatography column (Cytiva, HisTrap HP, 17524802) on an fast protein liquid chromatography (FPLC) system. The protein of interest was eluted with buffer B (20 mM HEPES, 500 mM NaCl, 500 mM imidazole, 1 mM DTT and 5% glycerol, pH 7.5). ARF1 was then purified by size-exclusion chromatography through a Superdex 75 Hiload 16/600 column (Cytiva 28-9893-33). The cDNA of human LBD-STING (139–379) was cloned into a pET-28 vector with an N-terminal His6-SUMO tag. LBD-STING was expressed in *Escherichia coli* BL21 (DE3) with 0.4 mM IPTG induction overnight at 16 °C. The cell pellet was lysed by sonication and purified on a Ni-NTA column in 50 mM Tris at pH 8.0, 350 mM NaCl, 20 mM imidazole and 0.5 mM phenylmethanesulfonyl fluoride (PMSF). The protein was eluted with buffer containing 50 mM Tris at pH 8.0, 350 mM NaCl and 300 mM imidazole, and then loaded onto a Superdex 75 HiLoad 16/600 column (Cytiva 28-9893-33) in PBS. The sample fractions were pooled and proteins were quantified by molar absorption measurements. All mutants were generated using a PCR-based technique with appropriate primers and confirmed by DNA sequencing. The mutant STING proteins were expressed and purified in the same way as the wild-type STING. His-tagged AP-1β 1–584, GST-tagged AP-1γ 1–595, AP-1μ 1–423 and AP-1σ 1–154 were cloned into a pST44 vector and referred to as AP-1 core. His-tagged AP-1β 1–584, GST-tagged AP-1γ 1–595, AP-1μ 1–142 and AP-1σ 1–154 were cloned into a pST44 vector and referred to as AP-1 ΔμCTD. The AP-1 core complex in the pST44 vector was expressed in BL21 (DE3) (Sigma-Aldrich, CMC0014). A single colony was inoculated in a culture flask with 400 ml LB with ampicillin (100 µg ml^−1^) and incubated with shaking (200 rpm, Infors-HT Multitron) at 37 °C overnight as preculture. Large-scale expression of the complex (8 l in total: 2 l in four 5-l Erlenmeyer flasks) was started the next day by pouring 100 ml of preculture into 2 l of Auto Induction Media Terrific Broth (Formedium, AIMTB0210) with ampicillin (100 µg ml^−1^). Flasks were incubated with shaking at 37 °C for 6 h, then incubated at 18 °C overnight. The cells were then collected by centrifugation (4,000*g*, 15 min). A cell pellet of 2 l expression culture was transferred in a Falcon 50-ml tube. The 2-l expression cells were solubilized in PBS with 1 mM DTT, 1 mM EDTA and 2% glycerol at pH 7.5, supplemented with AEBSF and cOmplete protease inhibitors, then lysed by sonication. The cell lysate was clarified by centrifugation followed by 0.45-µm filtration. The supernatant was first purified on a glutathione sepharose 4B 5-ml column (Cytiva 28401748) on a FPLC system (Cytiva Aktä Pure). After TEV cleavage at 4 °C overnight in a 3,500 molecular weight cut-off dialysis tubing against PBS with 5% glycerol at pH 7.5, the sample was passed through a Superose 6 HiLoad 16/600 size-exclusion chromatography column (Cytiva 29323952) equilibrated in PBS with 2% glycerol at pH 7.5, to remove the TEV protease as well as free GST. When used for GST pull-down, TEV cleavage was skipped and the pool of GST-AP-1 eluate was directly loaded on the size-exclusion chromatography column. AP-1 ΔμCTD was expressed and purified in the same way as AP-1 core.

### In vitro phosphorylation of SUMO–STING 139–379 by TBK1

The recombinant SUMO–STING stock protein was diluted in assay buffer containing 20 mM Tris, 25 mM MgCl_2_, 2 mM EDTA, 4 mM EGTA and 1 mM DTT at pH 7.5, supplemented with phosphatase inhibitor cocktails and protease inhibitors. The pH was controlled before and after addition in the sample of 10 mM ATP. TBK1 (MRC PPU Reagents, DU12469) was added at a ratio of 1:20 (w/w) TBK1:STING. The reaction was performed at 4 °C overnight. The sample was loaded on a Superdex 200 Increase 10/300 GL size-exclusion chromatography column (Cytiva, 28990944) to purify phosphorylated SUMO–STING from the other reagents. The phosphorylation assay was monitored by liquid chromatography electrospray ionization mass spectrometry(LC/ESI-MS).

### GST pull-down assay

For AP-1–ARF1 pull-down, 30 μg GST-tagged AP-1 complex, 10 μg ARF1 and 10 μg LBD-STING were mixed together or individually in 40 μl pull-down buffer (PBS supplemented with 2 mM MgCl_2_, 1 mM GTP and 2 mM TCEP). The mixture was incubated overnight on ice. Thirty microlitres of glutathione sepharose beads (Cytiva) were incubated with the mixture for 30 min at 4 °C. Excess proteins were washed off the beads using 200 μl pull-down buffer each time for four times. Twenty microlitres of 5× SDS loading buffer was added to the resin and the mixture was boiled for 5 min. The samples were then centrifuged briefly. Five microlitres of supernatant was analysed by SDS–PAGE. The protein bands were visualized by Coomassie blue staining. For AP-1 ΔμCTD pull-down, 30 μg GST-tagged AP-1 ΔμCTD complex and 10 μg LBD-STING were mixed together or individually in 40 μl PBS supplemented with 2 mM TCEP. The mixture was incubated overnight on ice. Thirty microlitres of glutathione sepharose beads (Cytiva) was incubated with the mixture for 30 min at 4 °C. Excess proteins were washed off the beads using 200 μl PBS each time for four times. Twenty microlitres of 5× SDS loading buffer was added to the resin and the mixture was boiled for 5 min. The samples were then centrifuged briefly. Five microlitres of supernatant was analysed by SDS–PAGE. The protein bands were visualized by Coomassie blue staining.

### Bio-layer interferometry

Bio-layer interferometry analyses were performed at 25 °C using a GatorPrime biosensor system (GatorBio) with streptavidin probes (GatorBio, 160002). STING, STING mutants or pSTING were mixed with biotin (EZ-Link-NHS-LC-LC-Biotin, Thermo Fisher Scientific) at a molar ratio of three biotin molecules to one STING and incubated at room temperature for 30 min, and then excess biotin was removed by using a desalting column (PD 10, Cytiva). Biotinylated STING (10 μg ml^−1^) was immobilized onto the streptavidin biosensor (GatorBio, 18-5019) for 1 min. The tips were washed with PBS buffer for 2 min to obtain a baseline reading, then the biosensors were dipped into wells containing the various concentrations of AP-1 ΔμCTD or its mutant for 5 min, which was followed by a 10-min buffer wash to allow the dissociation of molecules from the sensor. Data analysis was performed with GraphPad PRISM 9 using a standard 1:1 binding model. Two independent experiments were performed for each sample.

### Cryo-EM data acquisition

Two milligrams of GST-tag cleaved AP-1 core complex was incubated with 2 mg ARF1 for 30 min at room temperature in PBS supplemented with 2 mM MgCl_2_, 1 mM GTP and 2 mM TCEP. Two milligrams of pSTING was then added, and the mixture was incubated on ice overnight. Excess ARF1 and pSTING were removed with a Superose 6 increase 10/300 GL column (Cytiva) in PBS. The AP-1–ARF1–pSTING complex fraction was collected and concentrated to 0.8 mg ml^−1^. Aliquots of 3 μl of AP-1–ARF1–pSTING complexes were loaded onto glow-discharged holey carbon grids (Electron Microscopy Sciences, Q250AR1.3, Quantifoil, Au, R 1.2/1.3, 300 mesh). Grids were blotted for 4 s and plunge-frozen in liquid ethane using a Vitrobot at 4 °C and with 100% humidity. Grids were screened for particle presence and ice quality on a TFS Glacios microscope (200 kV), and the best grids were transferred to TFS Titan Krios G4. Cryo-EM data were collected using a TFS Titan Krios G4 transmission electron microscope (TEM), equipped with a Cold-FEG on a Falcon IV detector in electron counting mode. Falcon IV gain references were collected just before data collection. Data were collected with TFS EPU v.2.12.1 using aberration-free image shift protocol (AFIS), recording eight micrographs per ice hole. Movies were recorded at a magnification of 270,000×, corresponding to the 0.45 Å pixel size at the specimen level, with defocus values ranging from −0.8 to −1.8 μm. Exposures were adjusted automatically to 60 e^−^  Å^−2^ total dose, resulting in an exposure time of approximately 3 s per movie. In total, 30,004 micrographs in EER format were collected.

### Cryo-EM data processing

Motion correction was performed on raw stacks without binning using the cryoSPARC implementation of motion correction. A total of 1,701,051 particles were template-based automatically picked and particles were binned by a factor of 4. Two rounds of two-dimensional (2D) classification were performed, resulting in a particle set of 539,684 particles. Two-dimensional classification of particles showed that the relative orientation between STING and AP-1 was highly variable. Selected particles resulting from the 2D classification were used for ab initio reconstruction. After two rounds of ab initio reconstruction, 326,772 particles were selected on the basis of STING densities. The particles were re-centred and re-extracted by a binning factor of 2. The particles were subjected to iterative CTF refinement and non-uniform refinement in cryoSPARC to 2.34 Å. The reported resolutions are based on the gold-standard Fourier shell correlation 0.143 criterion. Local-resolution variations were estimated using cryoSPARC.

### Model building and refinement

The AP-1–ARF1–pSTING model was generated using a published AP-1–ARF1–tetherin Nef structure (PDB 6DFF). The AP-1–ARF1 model after removing the tetherin Nef ligand was docked into the cryo-EM map in Chimera and fine-tuned by manual adjustment with Coot. The pSTING tail was docked against the cryo-EM map in Coot and the whole model was refined in PHENIX. Several loop regions of AP-1, ARF1 and pSTING were manually adjusted to fit into the map using Coot. The model was refined in real space again in PHENIX. All structure figures were made using UCSF Chimera, UCSF ChimeraX and PyMOL.

### Reporting summary

Further information on research design is available in the Nature Research Reporting Summary linked to this article.

## Online content

Any methods, additional references, Nature Research reporting summaries, source data, extended data, supplementary information, acknowledgements, peer review information; details of author contributions and competing interests; and statements of data and code availability are available at 10.1038/s41586-022-05354-0.

### Supplementary information

Supplementary Figure 1 Uncropped gels.

Reporting Summary

Supplementary Table 1 Oligonucleotide sequences.

Supplementary Table 2 A list of antibodies used in the study.

### Source data

Source Data Figs. 1–3 and Extended Data Figs. 1,3, 4, 5, 6 and 9

## Figures and Tables

**Extended Data Table 1:**
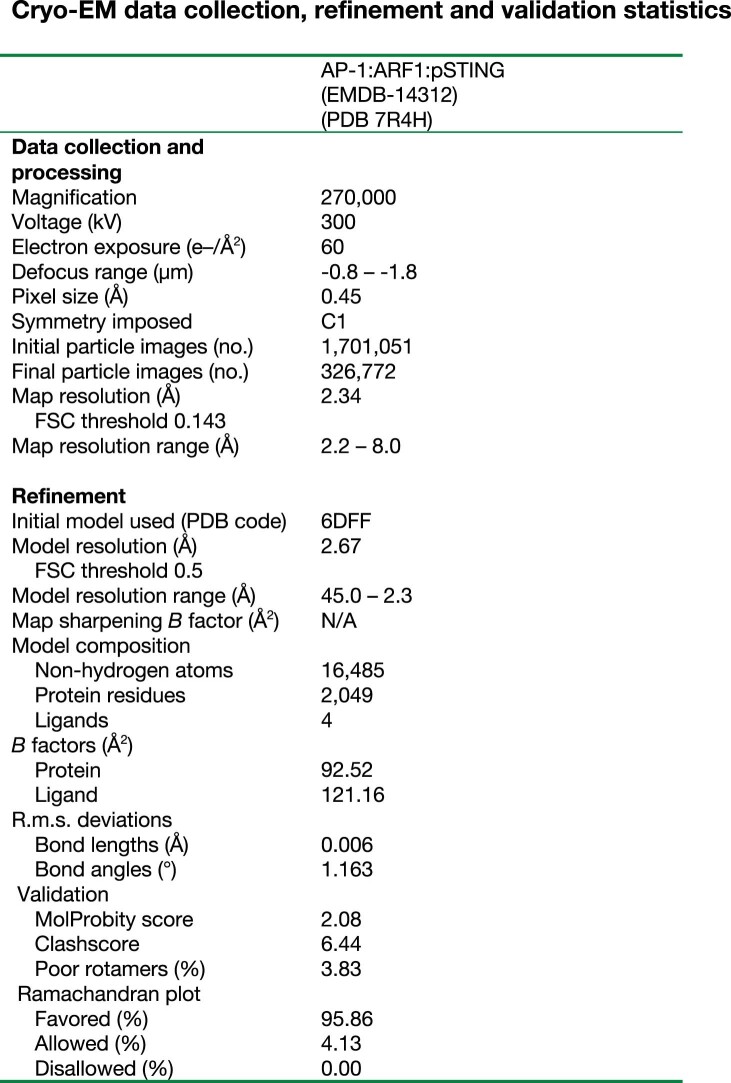
Cryogenic-electron microscopy data summary table

Details of cryo-EM data collection, refinement and validation statistics.

## Data Availability

Full scans for all western blots and the in-gel fluorescence images are provided in Supplementary Fig. 1. The 3D cryo-EM density map is deposited into the Electron Microscopy Data Bank under accession number EMD-14312. The coordinate is deposited in the PDB with accession number 7R4H. Source data are provided with this paper.
