# Supplementary material for: Clathrin-associated AP-1 controls termination of STING signalling
Source: Nature. 2022 Oct 19;610(7933):761–7. doi: 10.1038/s41586-022-05354-0 (PMC9605868; doi:10.1038/s41586-022-05354-0)
Supplement: Supplementary file 1 — Uncropped gels. [file 41586_2022_5354_MOESM1_ESM.docx]

**Clathrin-associated AP-1 controls termination of STING signalling**

Ying Liu^1,4^, Pengbiao Xu^1,4^, Sophie Rivara^1,4^, Chong Liu^1^, Jonathan Ricci^1^, Xuefeng Ren^2^, James H. Hurley^2,3^ & Andrea Ablasser^1,^*

^1^ Global Health Institute, Swiss Federal Institute of Technology Lausanne (EPFL), Lausanne, Switzerland, ^2^ Department of Molecular and Cell Biology and California Institute for Quantitative Biosciences, University of California, Berkeley, Berkeley, USA, ^3^ Helen Wills Neuroscience Institute, University of California, Berkeley, Berkeley, USA;

^4^ These authors contributed equally: Ying Liu, Pengbiao Xu, Sophie Rivara

* email: [andrea.ablasser@epfl.ch](mailto:andrea.ablasser@epfl.ch)

**Table of Contents:**

Supplementary Fig. 1

Source Data to Fig. 1 page 2-3

Source Data to Fig. 2 page 4-5

Source Data to Fig. 3 page 6-7

Source Data to Fig. 4 page 8

Source Data to Extended Data Fig. 3 page 9

Source Data to Extended Data Fig. 4 page 10

Source Data to Extended Data Fig. 5 page 11-12

Source Data to Extended Data Fig. 6 page 13-14

Source Data to Extended Data Fig. 7 page 15

Source Data to Extended Data Fig. 10 page 16

Supplementary Table S1 | Oligonucleotide sequences

Supplementary Table S2 | Antibody list

**Source data to Fig. 1a, f | Uncropped gels**

**
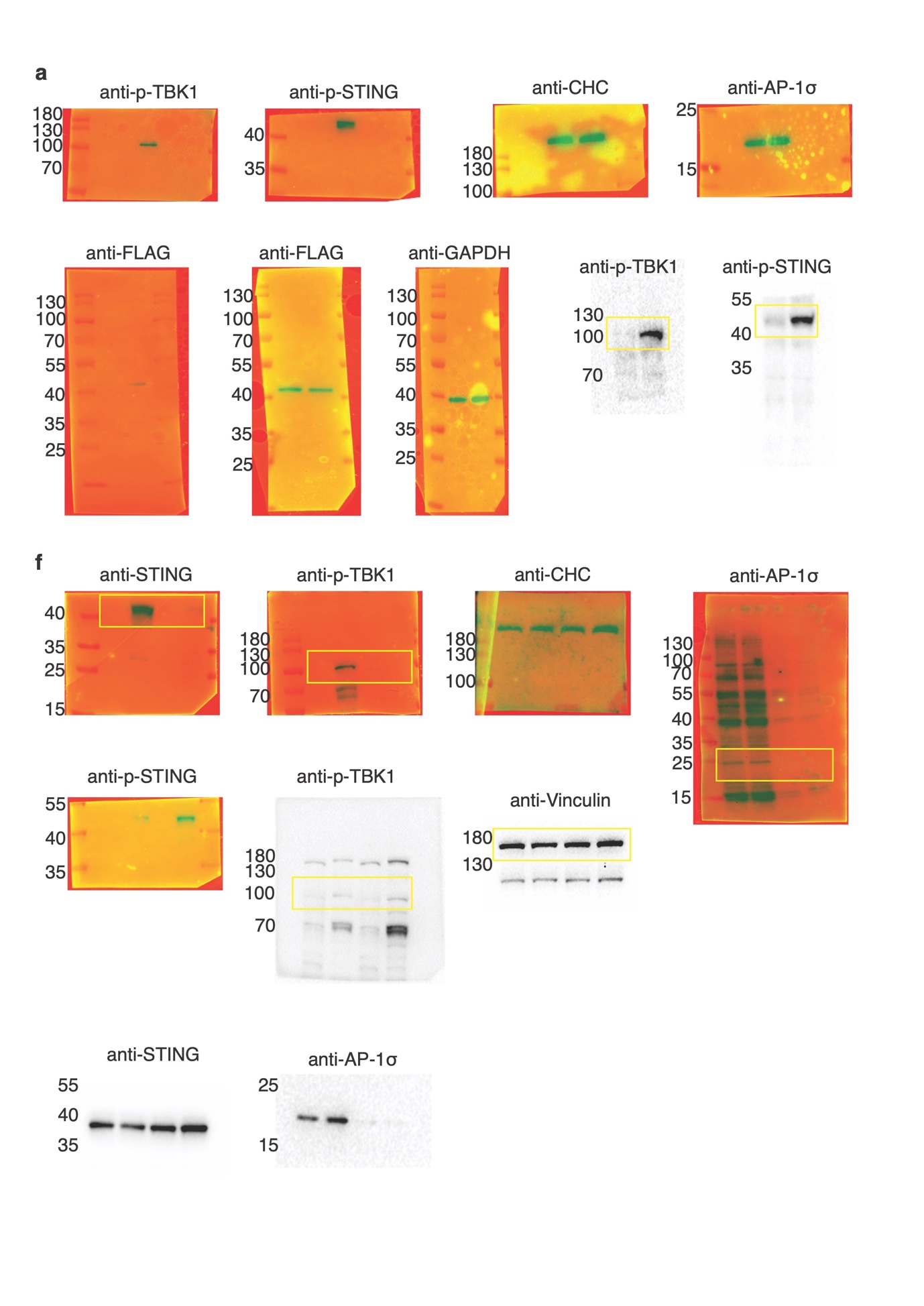
**

**Source data to Fig. 1h | Uncropped gels**

**
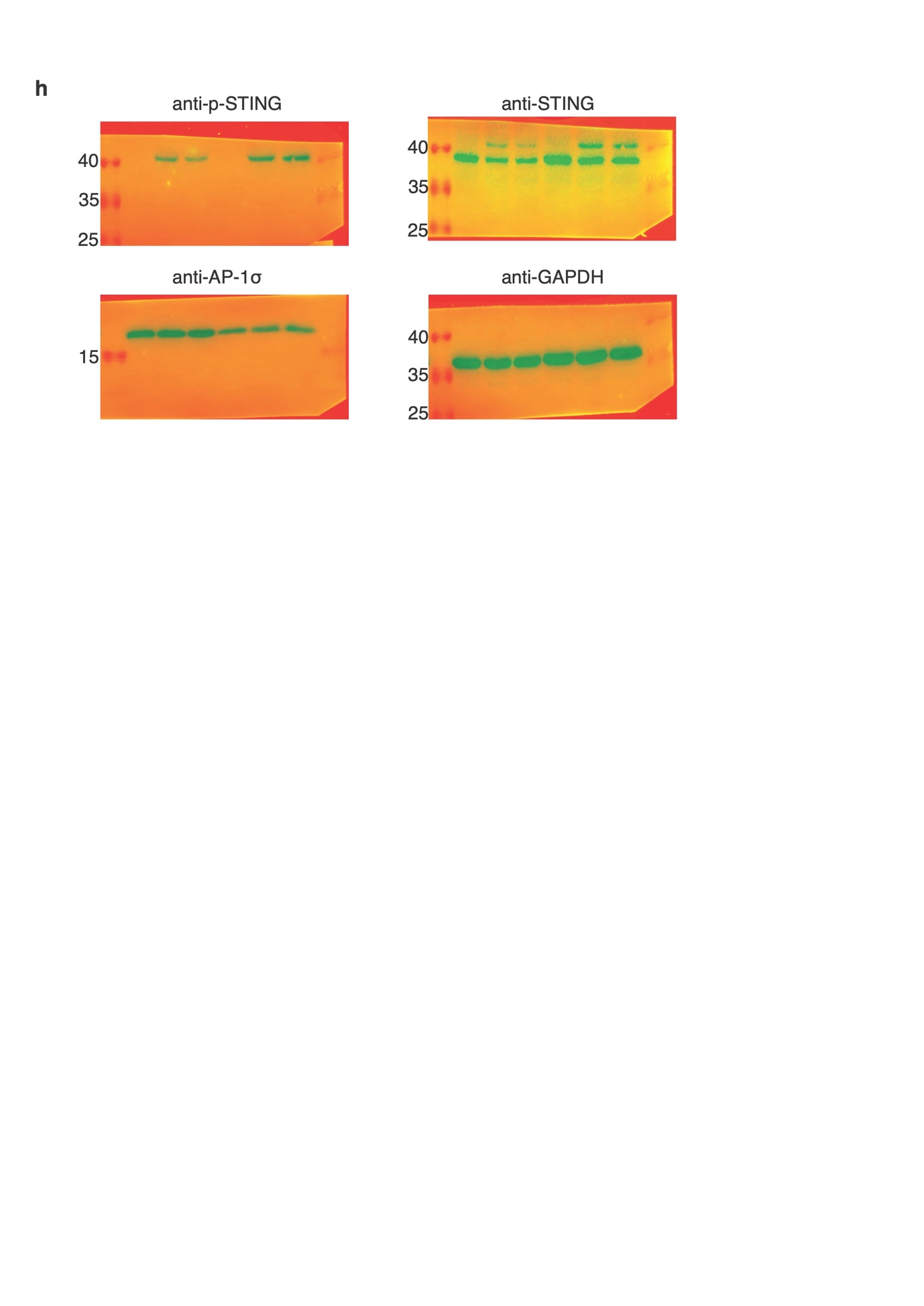
**

**Source data to Fig. 2b | Uncropped gels**

**
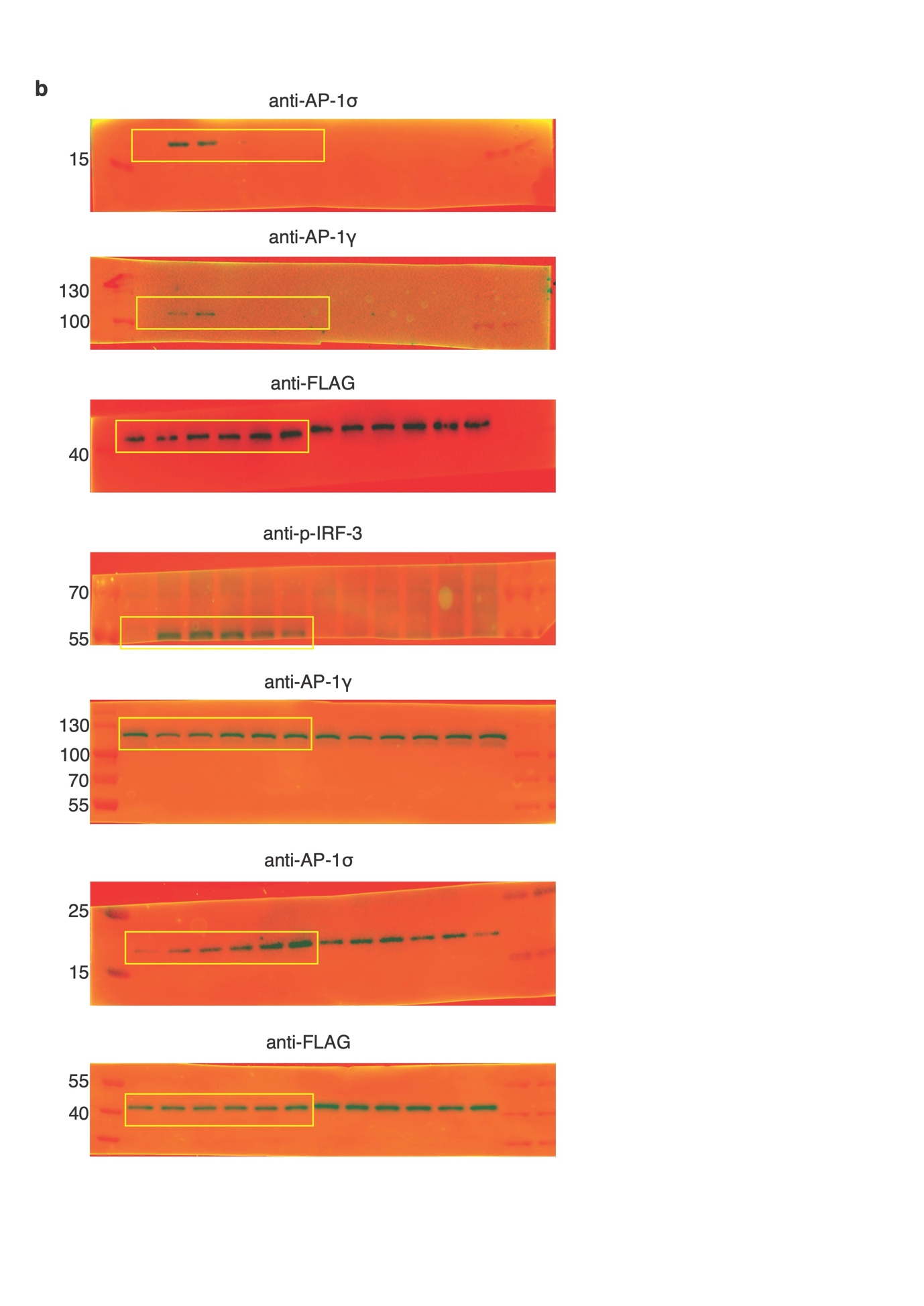
**

**Source data to Fig. 2c, d, f | Uncropped gels**

**
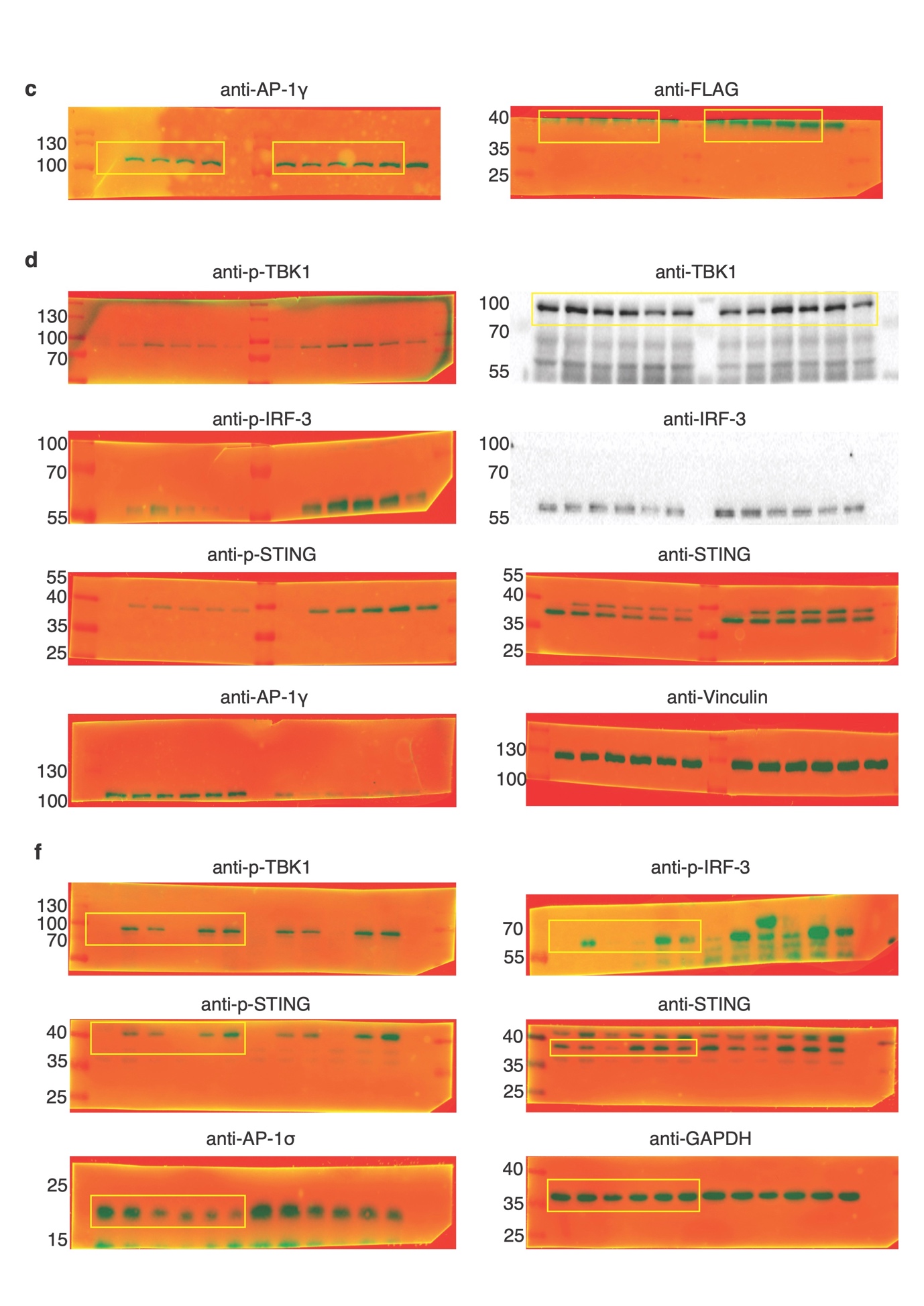
**

**Source data to Fig. 3b, c, d, e | Uncropped gels**

**
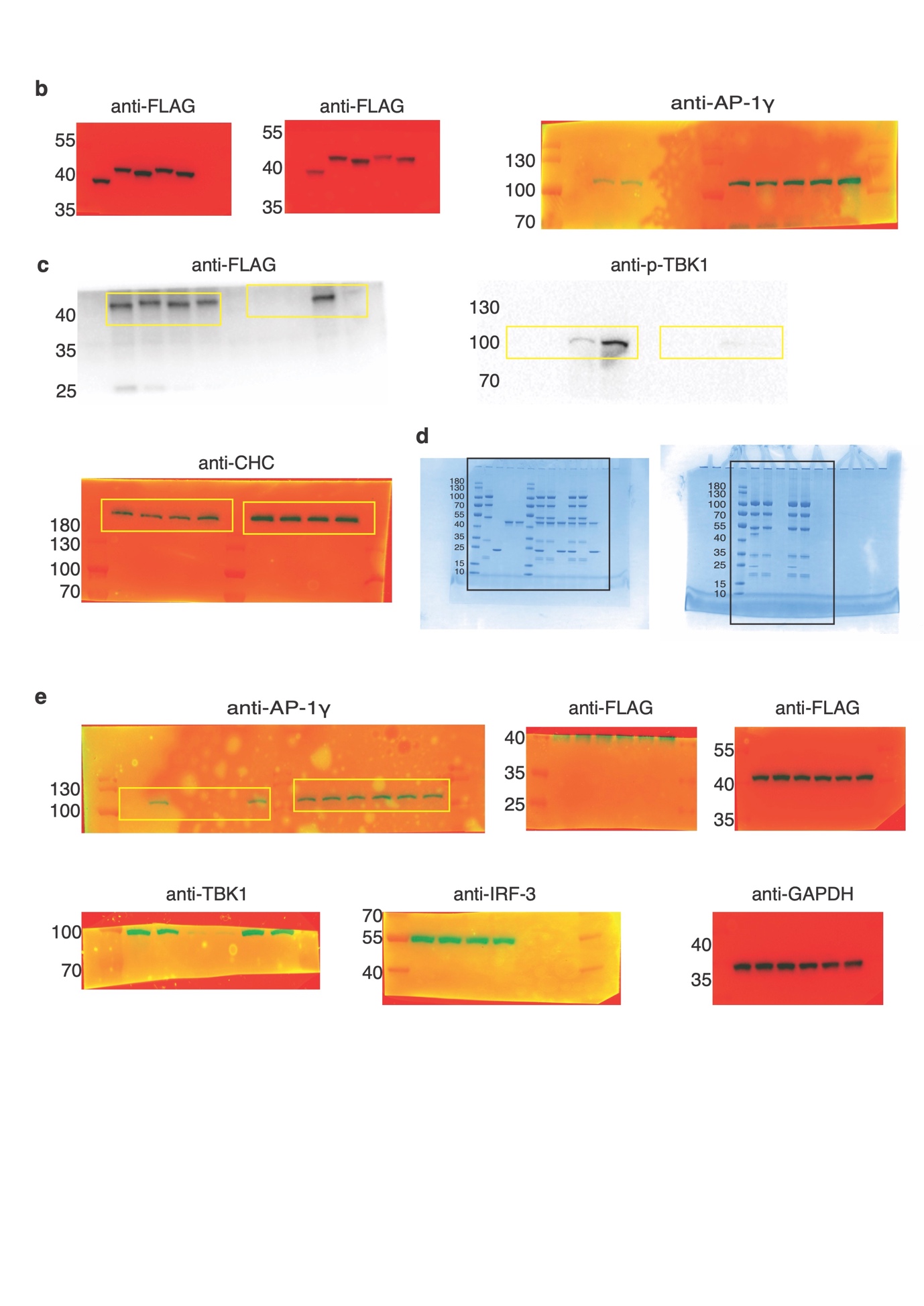
**

**Source data to Fig. 3f | Uncropped gels**

**
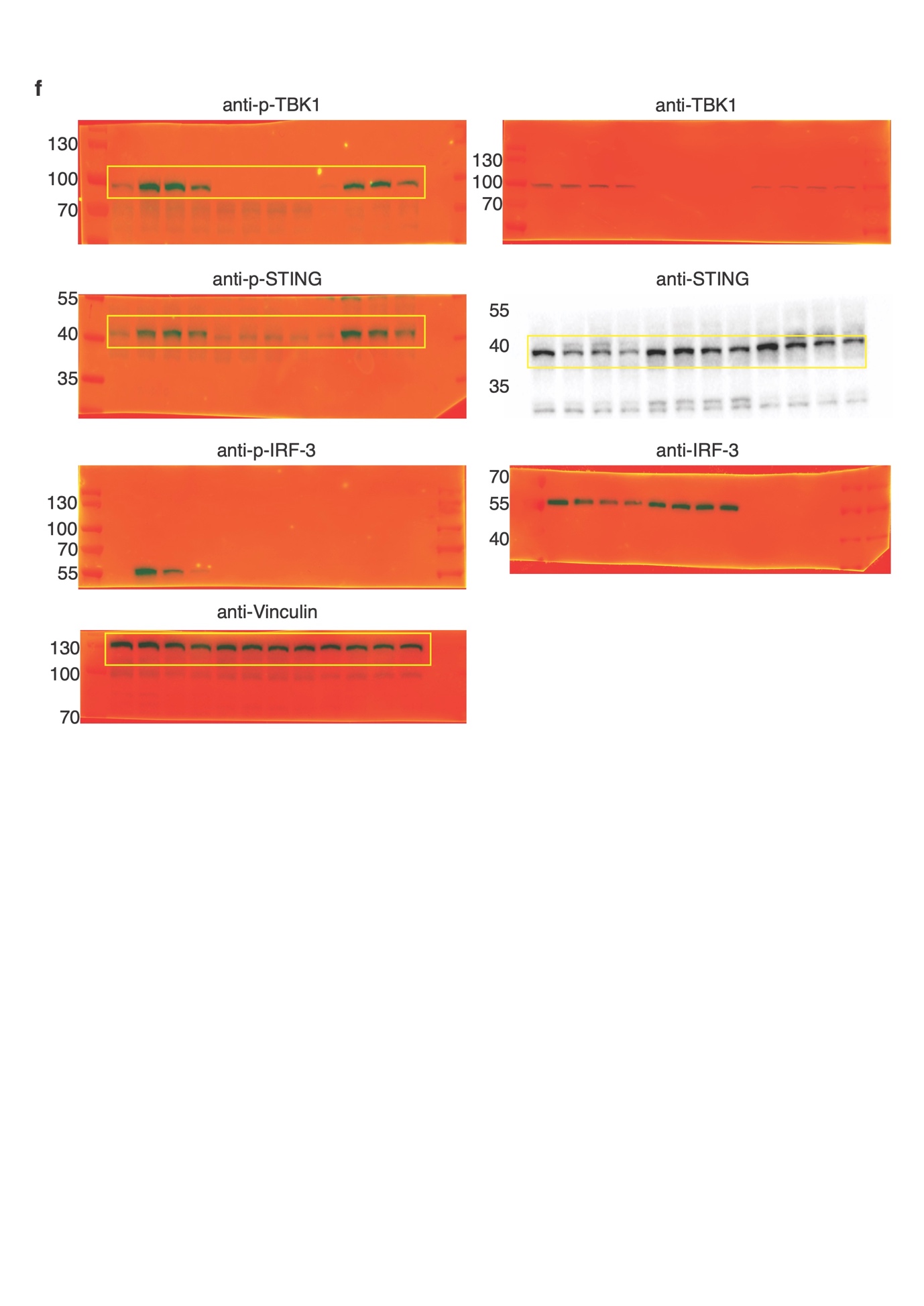
**

**Source data to Fig. 4g, h, i | Uncropped gels**

**
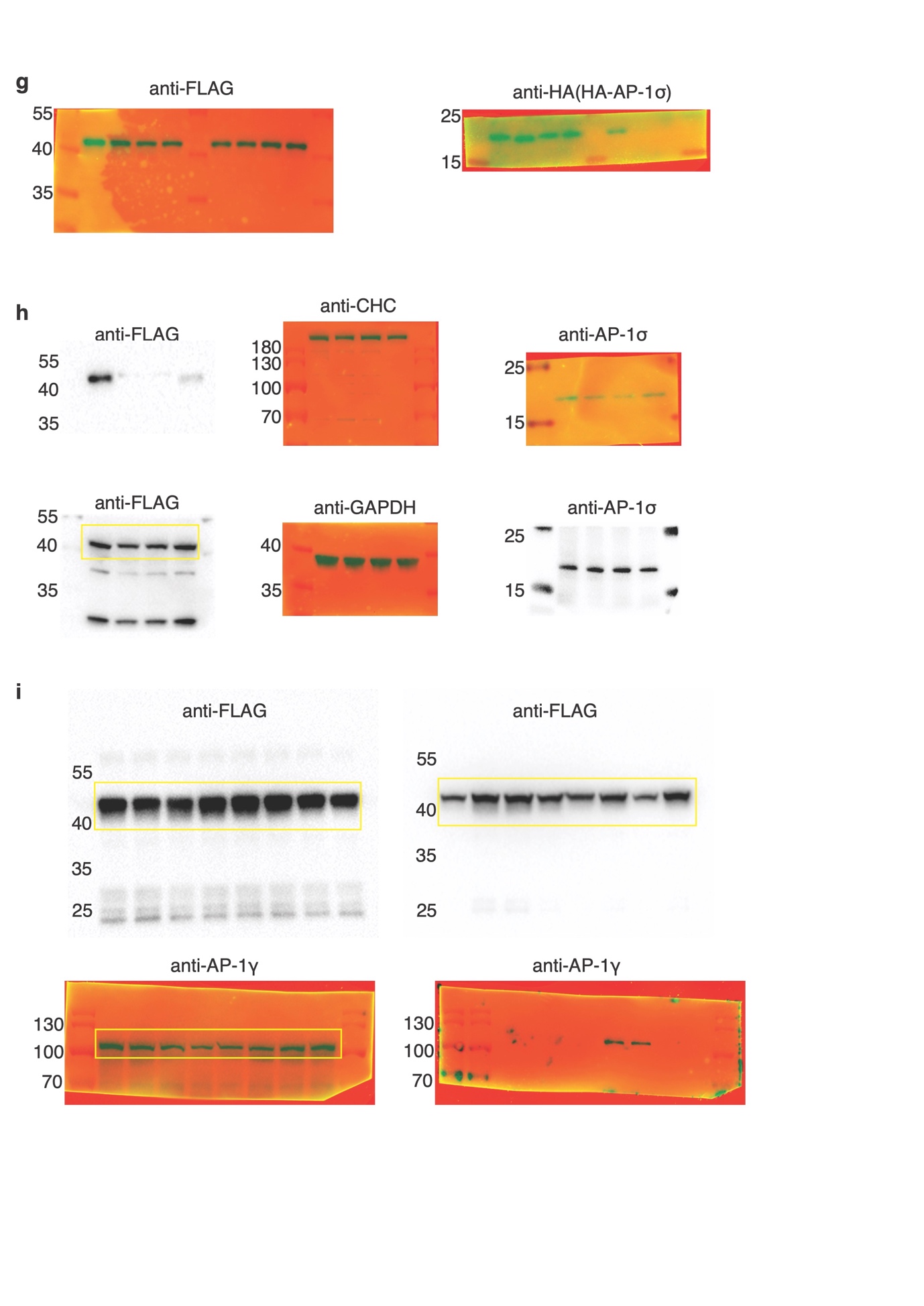
**

**Source data to Extended Data Fig. 3a, c, d | Uncropped gels**

**
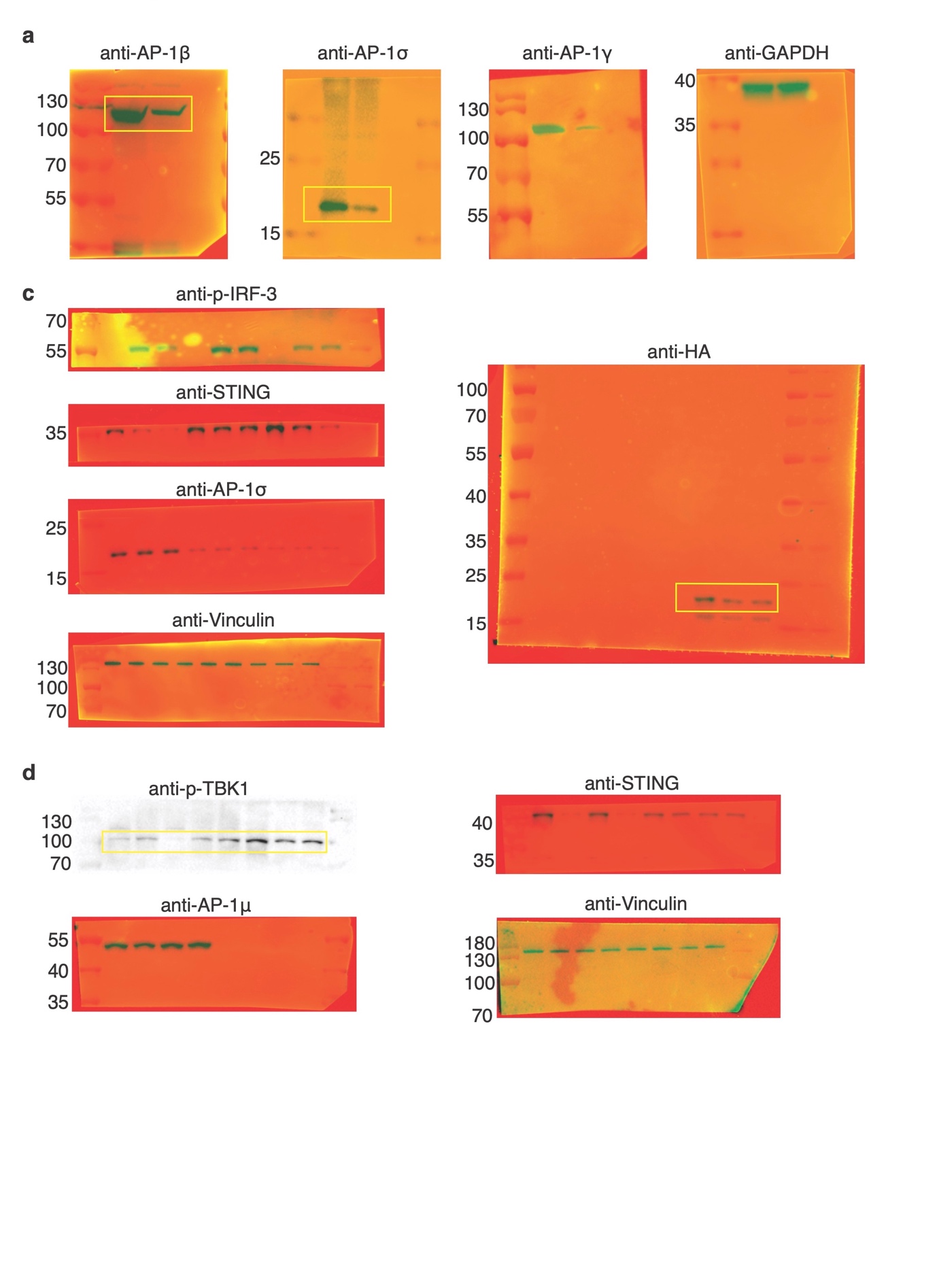
**

**Source data to Extended Data Fig. 4a, b, c, d | Uncropped gels**

**
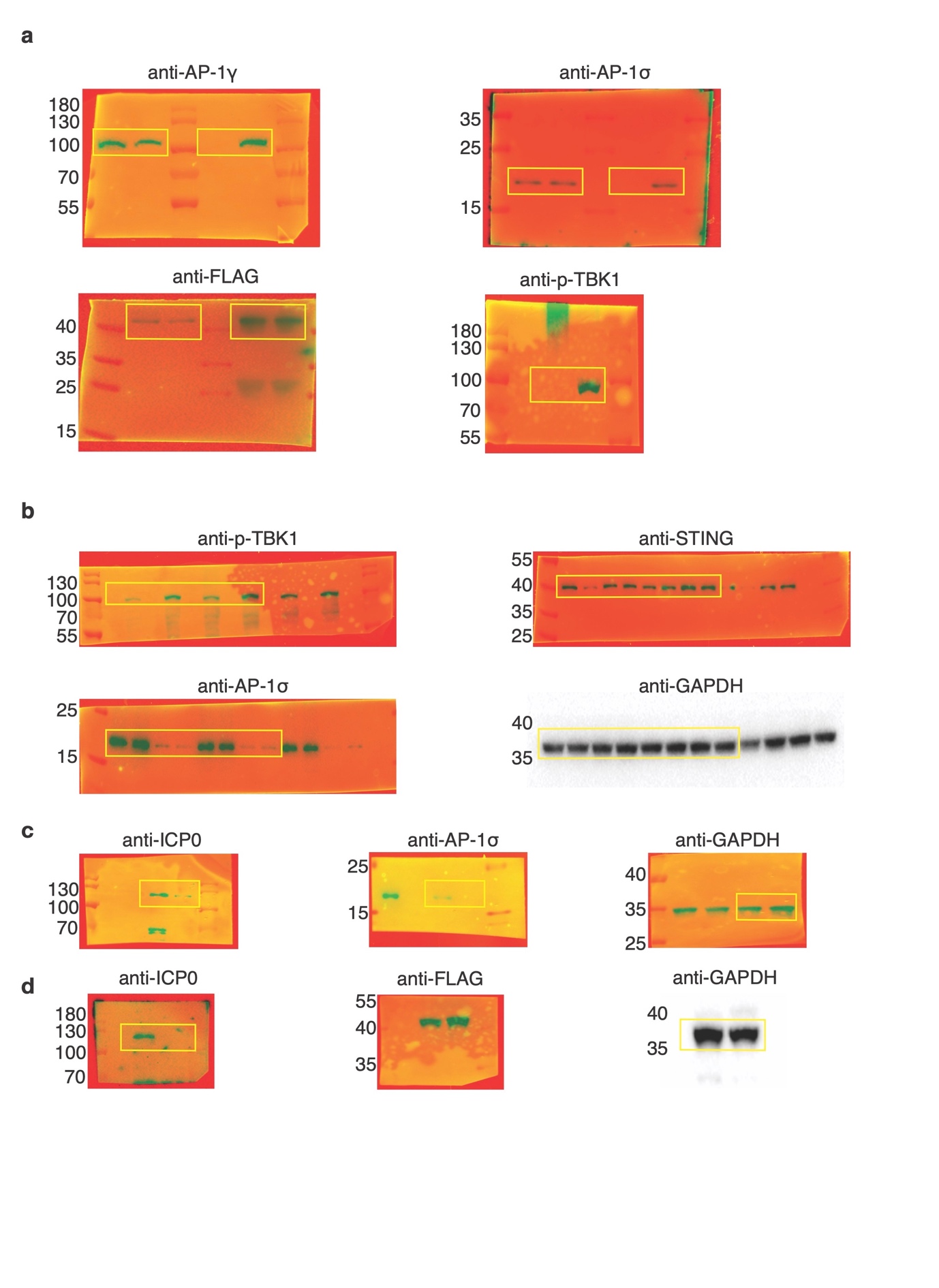
**

**Source data to Extended Data Fig. 5a, b | Uncropped gels**

**
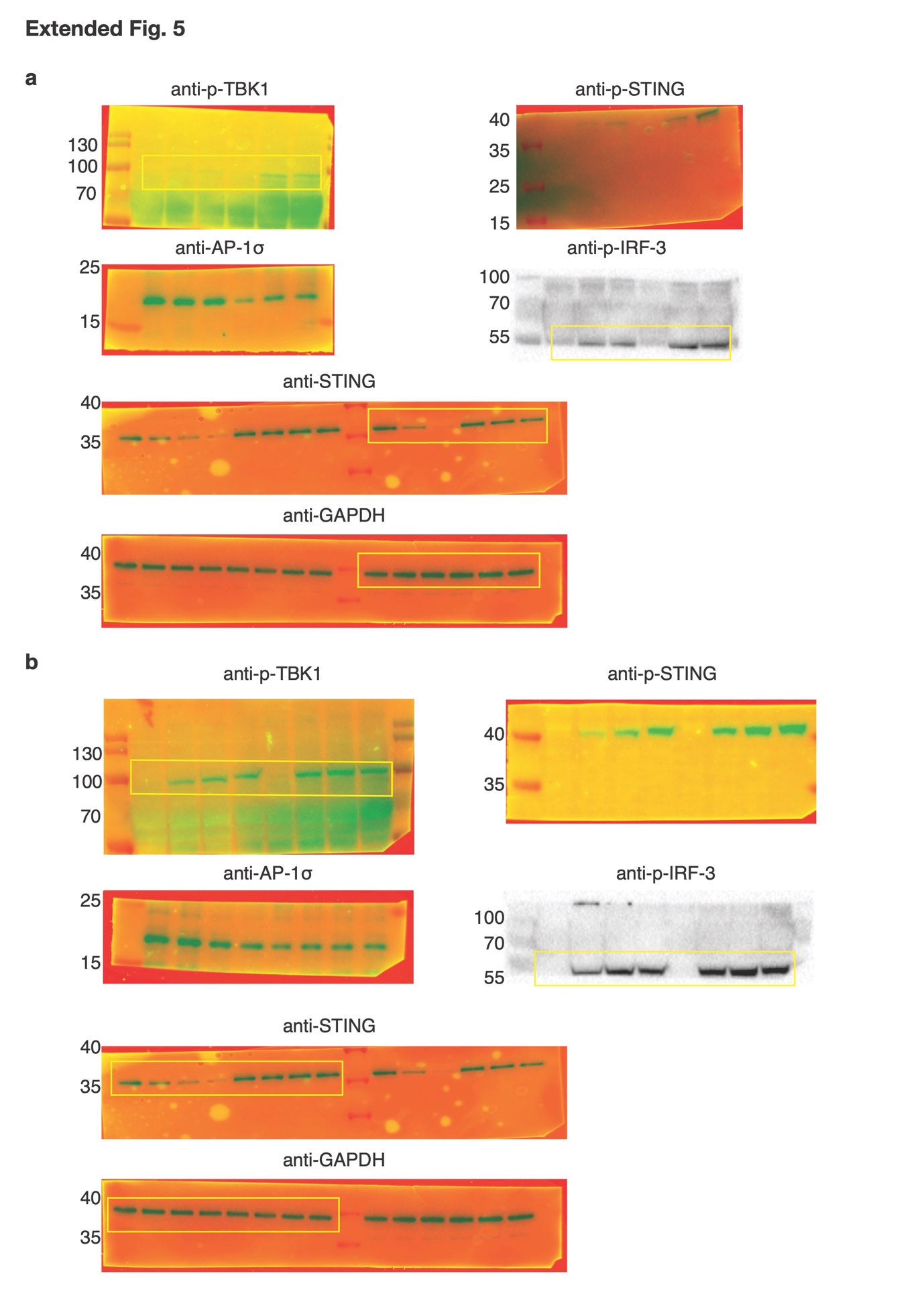
**

**Source data to Extended Data Fig. 5d, f, h, j | Uncropped gels**

**
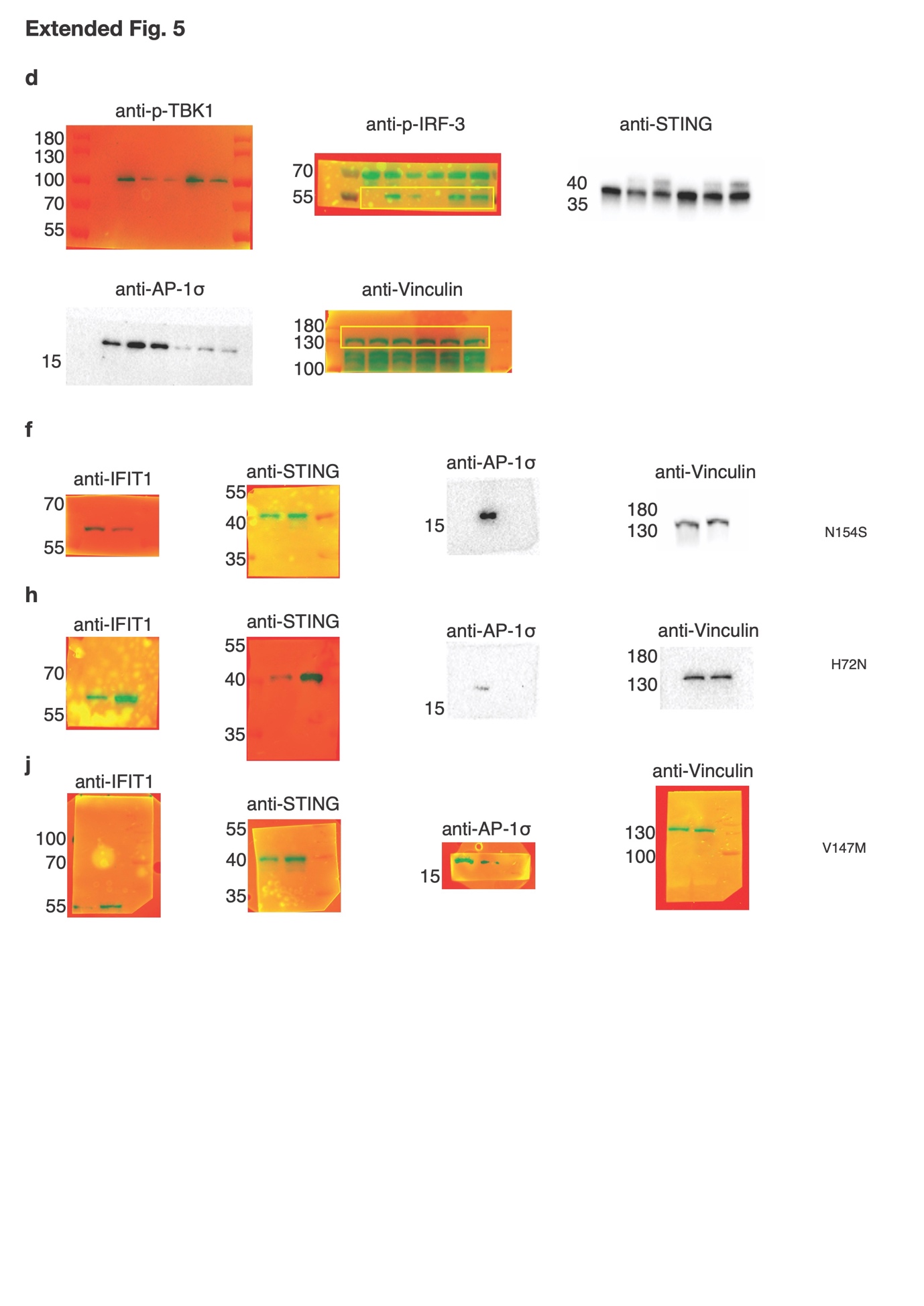
**

**Source data to Extended Data Fig. 6a, b, c | Uncropped gels**

**
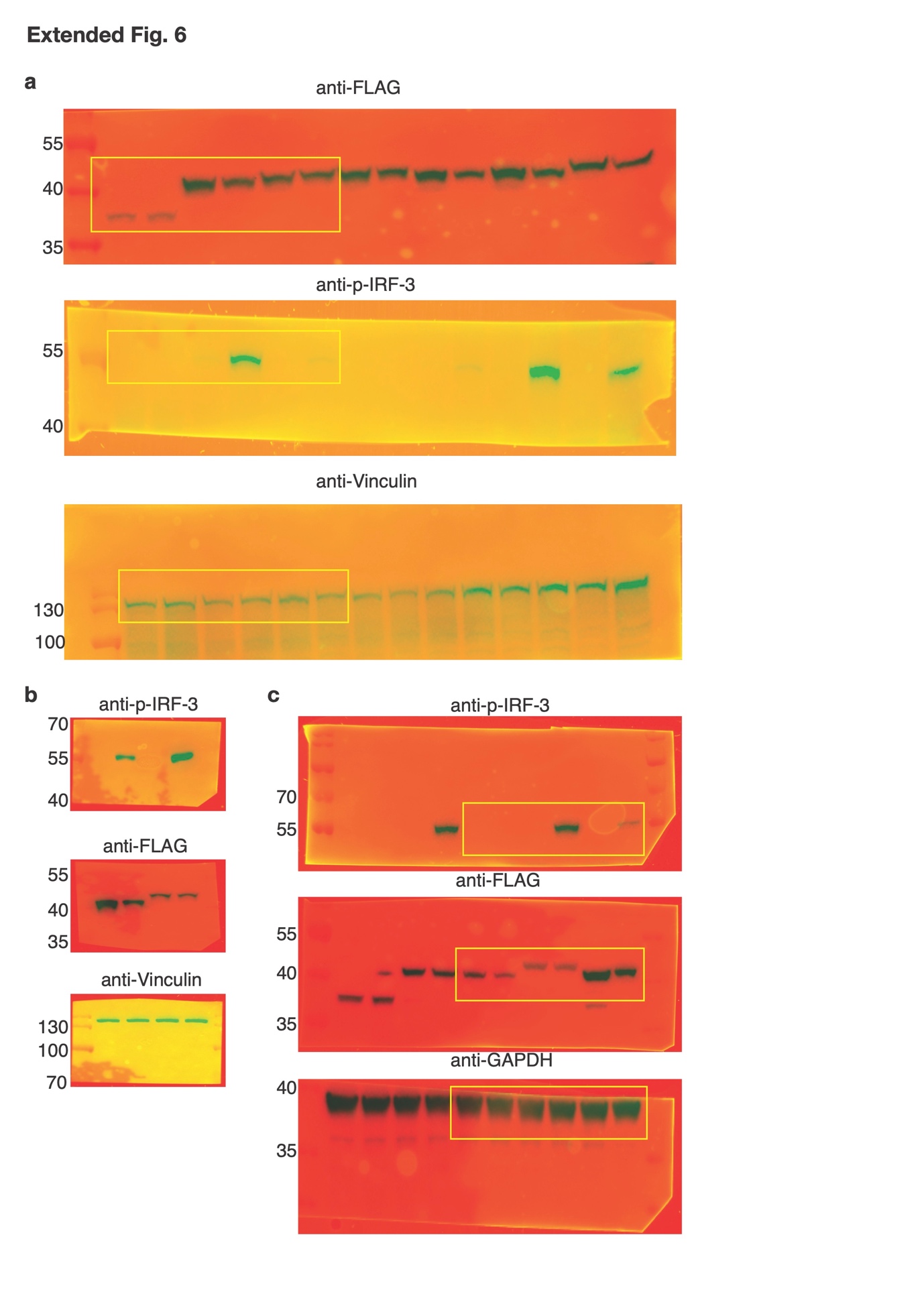
**

**Source data to Extended Data Fig. 6d, e, g | Uncropped gels**

**
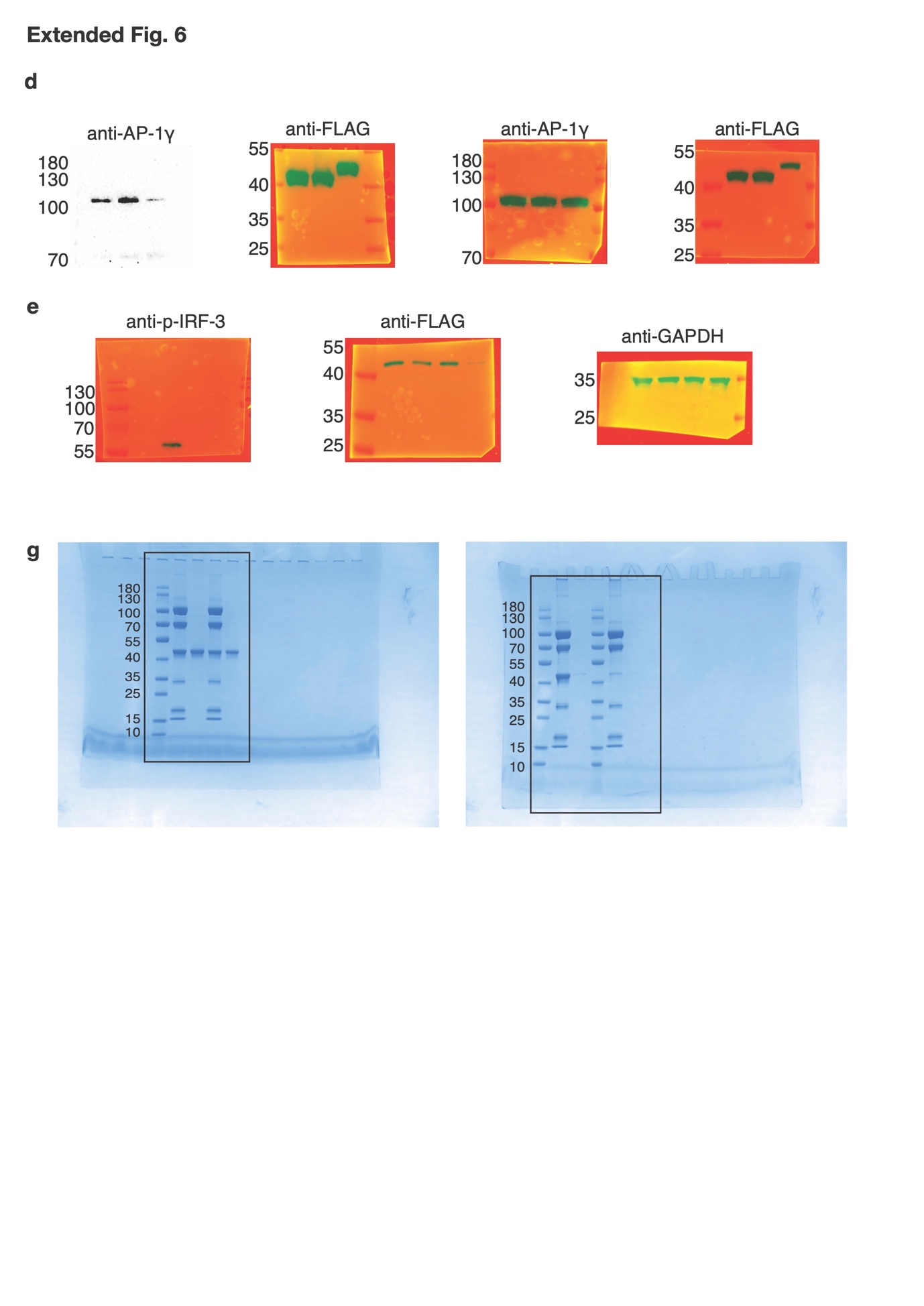
**

**Source data to Extended Data Fig. 7a, b, c | Uncropped gels**

**
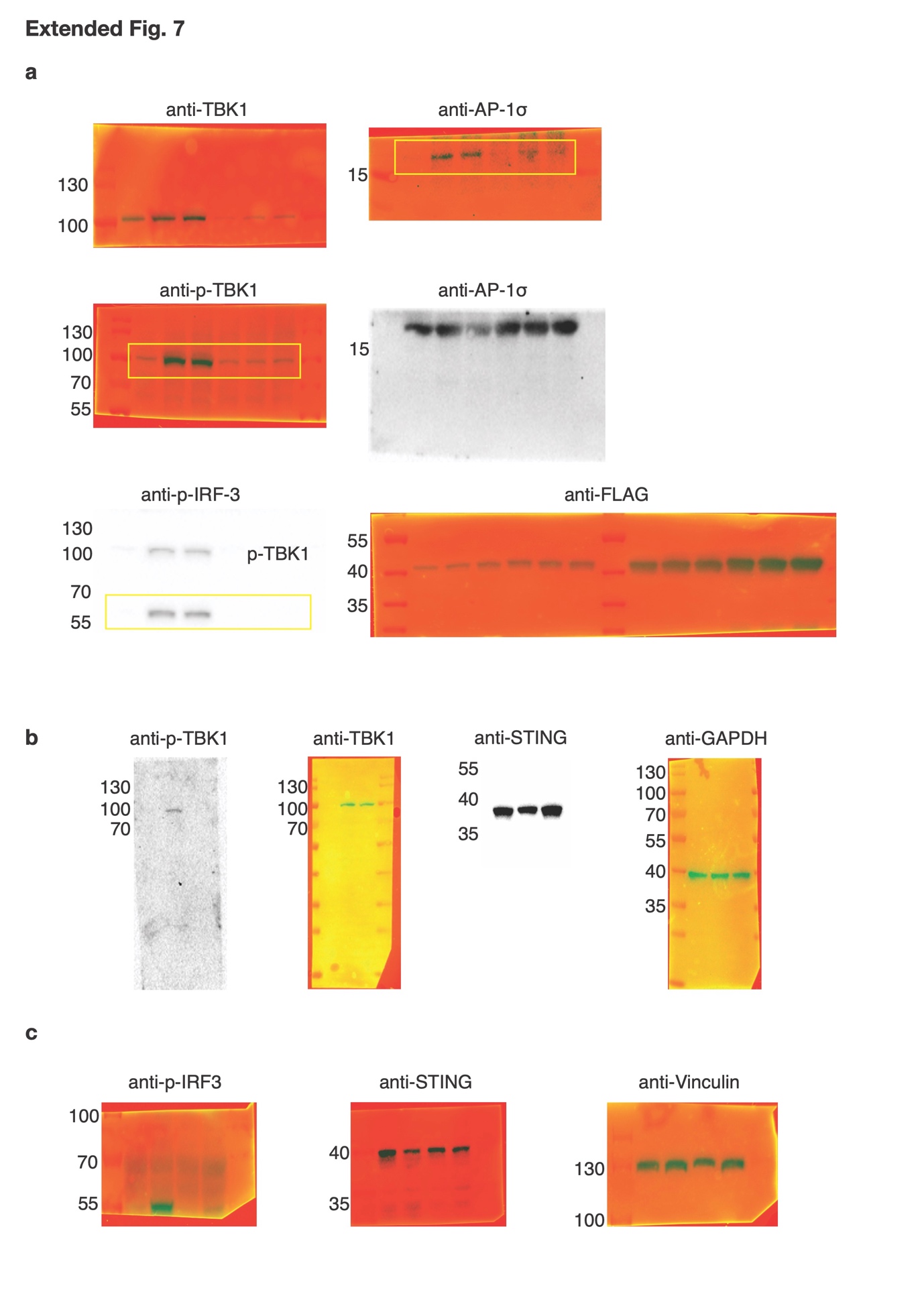
**

**Source data to Extended Data Fig. 10c | Uncropped gels**

**
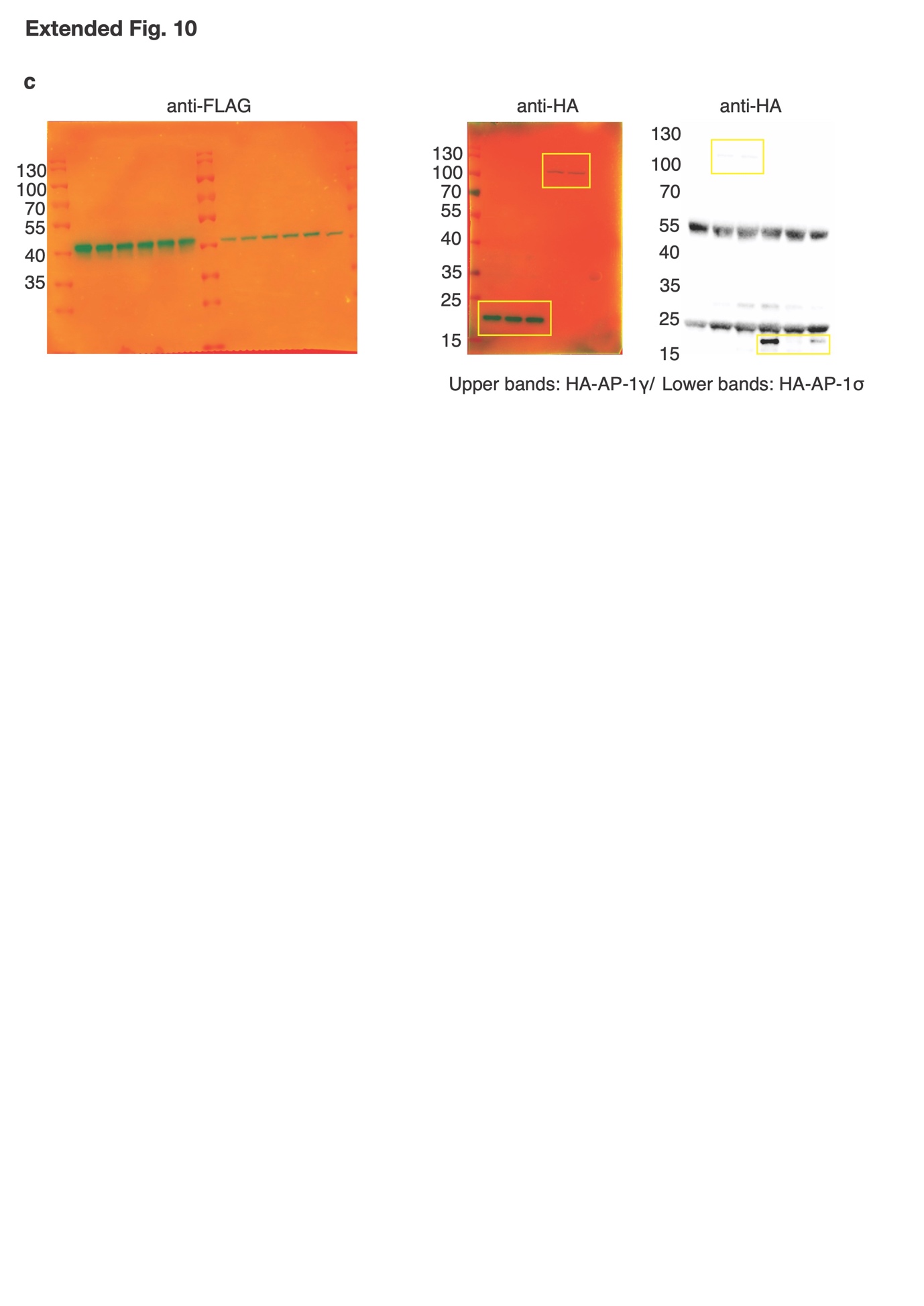
**
