# Supplementary material for: Clathrin-associated AP-1 controls termination of STING signalling
Source: Nature. 2022 Oct 19;610(7933):761–7. doi: 10.1038/s41586-022-05354-0 (PMC9605868; doi:10.1038/s41586-022-05354-0)
Supplement: Supplementary file 2 — Reporting Summary [file 41586_2022_5354_MOESM2_ESM.pdf]

nature portfolio

Corresponding author(s): Andrea Ablasser

Last updated by author(s): 02.09.2022

Reporting Summary

Nature Portfolio wishes to improve the reproducibility of the work that we publish. This form provides structure for consistency and transparency in reporting. For further information on Nature Portfolio policies, see our Editorial Policies and the Editorial Policy Checklist.

Statistics

For all statistical analyses, confirm that the following items are present in the figure legend, table legend, main text, or Methods section.

n/a	Confirmed	
☐	☒	The exact sample size (*n*) for each experimental group/condition, given as a discrete number and unit of measurement
☐	☒	A statement on whether measurements were taken from distinct samples or whether the same sample was measured repeatedly
☐	☒	The statistical test(s) used AND whether they are one- or two-sided<br>*Only common tests should be described solely by name; describe more complex techniques in the Methods section.*
☒	☐	A description of all covariates tested
☐	☒	A description of any assumptions or corrections, such as tests of normality and adjustment for multiple comparisons
☐	☒	A full description of the statistical parameters including central tendency (e.g. means) or other basic estimates (e.g. regression coefficient) AND variation (e.g. standard deviation) or associated estimates of uncertainty (e.g. confidence intervals)
☐	☒	For null hypothesis testing, the test statistic (e.g. *F*, *t*, *r*) with confidence intervals, effect sizes, degrees of freedom and *P* value noted<br>*Give P values as exact values whenever suitable.*
☒	☐	For Bayesian analysis, information on the choice of priors and Markov chain Monte Carlo settings
☒	☐	For hierarchical and complex designs, identification of the appropriate level for tests and full reporting of outcomes
☒	☐	Estimates of effect sizes (e.g. Cohen's *d*, Pearson's *r*), indicating how they were calculated

Our web collection on statistics for biologists contains articles on many of the points above.

Software and code

Policy information about availability of computer code

Data collection	Western blot and coomassie staining data were collected using CHemiDox XRS Biorad Imger and Image Lab Software (v6.1). RT-qPCR data were acquired using QuantStuido 7 Real-Time PCR system (Thermo Fisher).<br>Microscopy data were collected using LAS-X software v3.5.7 (for the Leica SP8 confocal microscope and STED microscope), PerkinElmer Harmony v4.9 (for the Operetta CLS microscope), ZEN Blue v3.4.91 (for the airyscan microscope).<br>Luminescence data were collected using TECAN SPARK 10M system.<br>Cryo-EM data were collected using EPU2.<br>EM data from CLEM were collected using a transmission electron microscope (FEI Company, Tecnai Spirit) with a digital camera (FEI Company, Eagle).
Data analysis	Western blot and coomassie staining data were analysed using ImageLab (v6.1) and quantified using Fiji (v2.3.0).<br>RT-qPCR data were analysed with GraphPad PRISM 9 (v9.3.1).<br>Microscopy data on the confocal and airyscan images were analyzed with Fiji (v2.3.0), using basic embedded tools and the BIOP JACoP plugin.<br>Microscopy data on the brightfield images from the Operetta microscopy were analyzed with PerkinElmer Harmony (v4.9).<br>Data tables obtained from PerkinElmer Harmony (v4.9) were then processed with KNIME (v4.3.2) for selecting results of interest and allowing batch calculations. Final results were then analysed and plotted using GraphPad PRISM 9 (v9.3.1).<br>All confocal and airyscan microscopy images were managed using OMERO.web (v5.11.0).<br>Cryo-EM structure was reconstructed by Cryosparc (v.3.3.1), Phenix (v.1.20rc2), Pymol (v.1.2), Coot (v. 0.8.9.3), UCSF Chimera (v.1.15), UCSF ChimeraX (v.1.2.5).<br>Correlation of EM and airyscan images for CLEM was performed using Adobe Photoshop software (v.22.5.8).<br>Deconvolution of STED images was performed using Huygens Remote Manager (v3.7).

For manuscripts utilizing custom algorithms or software that are central to the research but not yet described in published literature, software must be made available to editors and reviewers. We strongly encourage code deposition in a community repository (e.g. GitHub). See the Nature Portfolio guidelines for submitting code & software for further information.

Data

Policy information about availability of data

All manuscripts must include a data availability statement. This statement should provide the following information, where applicable:

- Accession codes, unique identifiers, or web links for publicly available datasets
- A description of any restrictions on data availability
- For clinical datasets or third party data, please ensure that the statement adheres to our policy

Full scans for all western blots and the in-gel fluorescence images are provided in Supplementary Figure 1. The three-dimensional cryo-EM density map is deposited into the Electron Microscopy Data Bank (EMDB) under accession number EMD-14312. The coordinate is deposited in the Protein Data Bank (PDB) with accession number 7R4H. Source data are available.

Field-specific reporting

Please select the one below that is the best fit for your research. If you are not sure, read the appropriate sections before making your selection.

☒ Life sciences ☐ Behavioural & social sciences ☐ Ecological, evolutionary & environmental sciences

For a reference copy of the document with all sections, see nature.com/documents/nr-reporting-summary-flat.pdf

Life sciences study design

All studies must disclose on these points even when the disclosure is negative.

Sample size	No statistical method was used to predetermine sample size. As mentioned in the legends, we have used at least three biological replicates per experiment, unless stated otherwise in the figure legends. This is consistent with previous studies and accounts for biological variability between distinct samples from cell lines.
Data exclusions	No data were excluded.
Replication	At least three repeats were performed, unless stated otherwise in the legends. The exact number (n) of biological replicates (cells, wells,...) are indicated in the legends. Experimental findings were consistent between replicates.
Randomization	All groups were randomly assigned.
Blinding	Data were not analyzed in a double-blinded manner.<br>Indeed microscopy data (for confocal, airyscan, STED, CLEM) was used for qualitative assessment and to exemplify cellular events. However, the only quantitative analysis performed on imaging was performed on the entire Operetta dataset using predefined fields of view sampling all wells similarly between conditions. The analysis pipeline was run on all obtained pictures with no pre-selection, nor data exclusion, nor adaptation of the pipeline between conditions, so that it can be considered unbiased between conditions.

Reporting for specific materials, systems and methods

We require information from authors about some types of materials, experimental systems and methods used in many studies. Here, indicate whether each material, system or method listed is relevant to your study. If you are not sure if a list item applies to your research, read the appropriate section before selecting a response.

Materials & experimental systems

n/a	Involved in the study	
☐	☒	Antibodies
☐	☒	Eukaryotic cell lines
☒	☐	Palaeontology and archaeology
☒	☐	Animals and other organisms
☒	☐	Human research participants
☒	☐	Clinical data
☒	☐	Dual use research of concern

Methods

n/a	Involved in the study	
☒	☐	ChIP-seq
☒	☐	Flow cytometry
☒	☐	MRI-based neuroimaging

Antibodies

Antibodies used	Primary antibodies used: mouse monoclonal anti-Vinculin (hVIN-1) (Sigma-Aldrich, V9264, immunoblot 1:5000), rabbit monoclonal anti-GAPDH (14C10) (Cell Signaling Technology, 2118, immunoblot 1:3000), mouse monoclonal anti-FLAG (M2) (Sigma-Aldrich, F1804, immunoblot 1:3000, IF 1:400), rabbit monoclonal anti-human phospho-STING (Ser366) (D7C3S) (Cell Signaling Technology, 19781, immunoblot 1:3000), rabbit monoclonal anti-phospho-TBK1/NAK (Ser172) (D52C2) (Cell Signaling Technology, 5483,

immunoblot 1:1000), rabbit monoclonal anti-phospho-IRF-3 (Ser 386) (EPR2346) (Abcam, ab76493, immunoblot 1:1000), rabbit monoclonal anti-TBK1/NAK (D1B4) (Cell Signaling Technology, 3504, immunoblot 1:1000), rabbit polyclonal anti-TMEM173/STING (Proteintech, 19851-1-AP, immunoblot 1:1000), rabbit monoclonal anti-IRF-3 (D6I4C) (Cell Signaling Technology, 11904, immunoblot 1:1000), rabbit monoclonal anti- Clathrin Heavy Chain (P1663) (Cell Signaling Technology, 2410, immunoblot 1:500, IF 1:100), mouse anti-Clathrin Heavy Chain Monoclonal Antibody (X22) (ThermoFisher, # MA1-065, IF 1:100), rabbit polyclonal anti-AP1S1 (Thermo Fisher, PA5-63913, immunoblot 1:1000), rabbit polyclonal anti-AP1G1 (Thermo Fisher, PA5-65290, immunoblot 1:1000), rabbit polyclonal anti-AP1B1 (Sigma-Aldrich, HPA065226, immunoblot 1:1000), rabbit polyclonal anti-AP1M1 (Proteintech, 12112-1-AP, immunoblot 1:1000), mouse monoclonal anti-HSV-1 ICP0 (11060) (Santa Cruz, sc-53090, immunoblot 1:500), mouse monoclonal anti-HA.11 Epitope Tag (16B12) (Biolegend, MMS-101R, immunoblot 1:2000). mouse monoclonal γ-Adaptin (AP1G1) (100/3) (Sigma-Aldrich, A4200, IF 1:100), mouse monoclonal EEA1 (E9Q6G) (Cell Signaling, 48453, IF 1:100), mouse monoclonal LAMP1 (H4A3) (Abcam, ab25630, IF 1:100), rabbit monoclonal anti-human phospho-STING (Ser366) (D8K6H) (Cell Signaling Technology, #40818, IF 1:100, STED 1:50), mouse monoclonal Rab7 (E9O7E) (Cell Signaling Technology, 95746, IF 1:100), sheep polyclonal human-TGN46 (BioRad, AHP500G, IF 1:200).
HRP-conjugated secondary antibodies used: Donkey anti-rabbit IgG (H+L)-HRP (Jackson ImmunoResearch, 711-036-152, immunoblot: 1:5000) and Donkey anti-mouse IgG (H+L)-HRP (Jackson ImmunoResearch, 715-036-151, immunoblot: 1:5000).
Fluorescence-conjugated secondary antibodies used: Goat anti-Mouse IgG2a Cross-Adsorbed Secondary Antibody, Alexa Fluor 647-conjugated (Invitrogen, A-21241, IF 1:800), Donkey anti-Sheep IgG (H+L) Cross-Adsorbed Secondary Antibody, Alexa Fluor 488-conjugated (Invitrogen, A-11015, IF 1:800), Goat anti-Rabbit IgG (H+L) Cross-Adsorbed Secondary Antibody, Alexa Fluor 568-conjugated (Invitrogen, A-11011, IF 1:800), Goat-Anti Rabbit-Atto647N (Hypermol,2318, IF 1:500).

Validation

For the primary antibodies used in this study, we relied on species and application validation as stated on the manufacturers' websites. Summary of those statements are displayed in the Supplementary table 2.

Eukaryotic cell lines

Policy information about cell lines

Cell line source(s)

HeLa (CCL-2) cells were obtained from Sigma-Aldrich. HEK 293T cells were a gift from Didier Trono (EPFL), originally purchased from ATCC (cat. no. SD-3515). HeLa STING KO cell line was a gift from Fabio Martinon (University of Lausanne) and were generated as described in the reference 50 of our paper. THP-1 cells and WI-38 cells were obtained from ATCC. HaCaT cells were obtained from CLS. Primary human alveolar epithelial cells (epithelial cells) were obtained from a commercial supplier (Cell Biologics). MEFs (µ1 KO cells and µ1 KO cells reconstituted with µ1A) were a gift from Peter Schu (University Medical Center Göttingen).

Authentication

Cells were frequently checked by their morphological features.

Mycoplasma contamination

All cell lines were tested to be mycoplasma-negative by PCR repeatlly.

Commonly misidentified lines
(See ICLAC register)

No commonly misidentified cell lines are used in this study.
